# Solid-Phase Synthetic Route to Multiple Derivatives of a Fundamental Peptide Unit

**DOI:** 10.3390/molecules15074961

**Published:** 2010-07-20

**Authors:** William L. Scott, Ziniu Zhou, Paweł Zajdel, Maciej Pawłowski, Martin J. O’Donnell

**Affiliations:** 1 Department of Chemistry and Chemical Biology, Indiana University-Purdue University Indianapolis, 402 N. Blackford Street, Indianapolis, IN 46202, USA; 2 Department of Medicinal Chemistry, Jagiellonian University Medical College, 9 Medyczna Street, 30-688 Kraków, Poland

**Keywords:** Distributed Drug Discovery (D3), fundamental peptide scaffold, biomimetics, solid-phase organic synthesis, combinatorial chemistry

## Abstract

Amino acids are Nature’s combinatorial building blocks. When substituted on both the amino and carboxyl sides they become the basic scaffold present in all peptides and proteins. We report a solid-phase synthetic route to large combinatorial variations of this fundamental scaffold, extending the variety of substituted biomimetic molecules available to successfully implement the Distributed Drug Discovery (D3) project. In a single solid-phase sequence, compatible with basic amine substituents, three-point variation is performed at the amino acid α-carbon and the amino and carboxyl functionalities.

## Introduction

The Distributed Drug Discovery (D3) project seeks to simultaneously educate and innovate while searching for drug leads for neglected diseases [1,2,3,4,5,6]. Central to this effort is the availability of simple, inexpensive and reproducible synthetic procedures providing access to large numbers of biomimetic molecules. To increase the diversity of molecule types available to D3 we decided to develop more flexible synthetic routes to derivatives of the peptide unit **1**, one of the most fundamental and inherently biomimetic scaffolds in nature. There are many examples of drugs or drug leads based on this scaffold. They arise from natural or unnatural amino acids modified at both the amino and carboxylic acid functionalities. These include the recently approved drug lacosamide (Vimpat^®^, **1a**) to treat epilepsy [7]. Special cases of **1** are **2** and **3**, in which R^3^ contains a basic amino group and n = 1 or 2. Compounds **3** have been shown to be selective binding agents for subclasses of 5-hydroxytryptamine (5-HT, serotonin) receptors [8]. This paper reports a flexible solid-phase synthetic route to many variations of R^1^, R^2^, and R^3^ on structures **2** and **3**. It will be the basis for future synthetic laboratories compatible with the D3 process.

**Figure 1:**
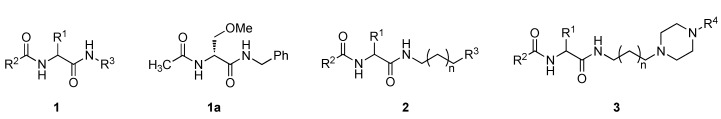
Important molecules containing the fundamental peptide unit **1**.

**Scheme 1:**
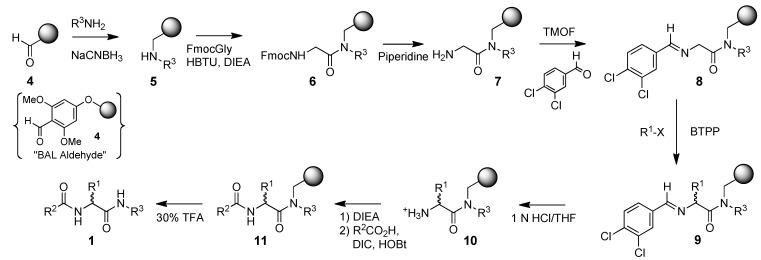
Synthetic route to amino- and carboxyl-substituted unnatural amino acids.

Compounds **1** can be readily synthesized on solid-phase using BAL type resins and naturally occurring amino acids [9,10]. For more extensive sets of derivatives, unnatural amino acid side chains R^1^ can be introduced during the course of the solid-phase synthesis utilizing the recently described chemistry outlined in Scheme 1 [6].

**Scheme 2:**
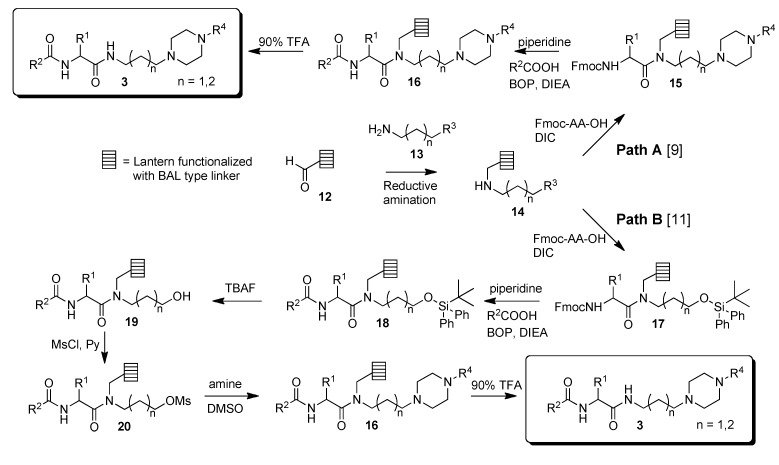
Synthetic pathways **A** and **B** to compounds **3**.

Compounds **3** are usually constructed from naturally occurring amino acids in which the amino group is acylated with a variety of carboxylic acids and the carboxyl group is amidated with amines containing an additional basic amino group. The solid-phase synthesis of **3** utilizes BAL-type linkers, “lantern” technology, and either of two synthetic routes (Scheme 2, Path A or B). While Path A provides a direct route to product, Path B (through intermediate **17**) permits greater flexibility by incorporation of many amines *after* attachment of the amino acid onto the BAL resin [11].

## Results and Discussion

**Scheme 3:**
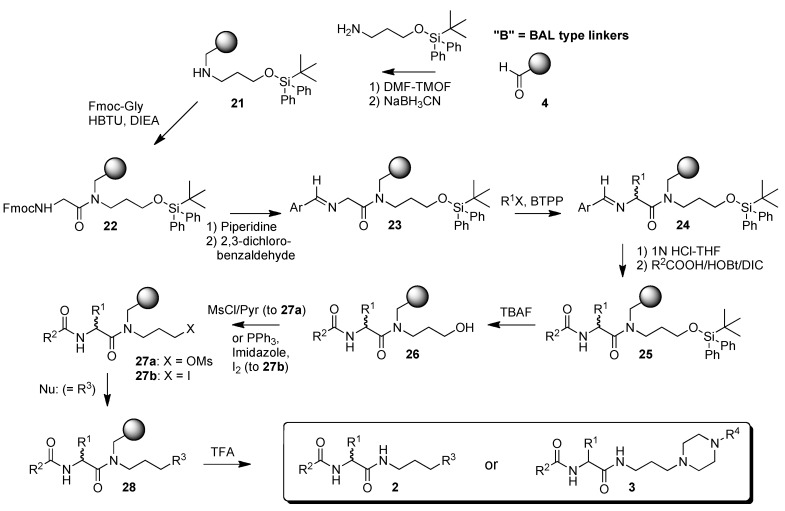
Synthesis of multiply-substituted **2** where R^3^ contains a nucleophilic site.

We sought to adapt the route shown in Scheme 1 to the synthesis of a more diverse set of compounds **2** and **3**, where R^1^ would possess both *R* and *S* configuration, not be restricted to naturally occurring amino acid side chains, and R^3^ in **2** contained basic amine functionality that could be readily varied. However, a key step in this synthesis would be the alkylation of intermediate **8** to **9** (Scheme 1), and any nucleophilic functionality present (such as the tertiary amines present in **3**) would likely lead to side reactions. Therefore, we decided to modify our chemistry to perform the alkylation on a derivative of key intermediate imine **8** that would be compatible with this alkylation chemistry and permit subsequent simple conversion to multiple derivatives **2** even if R^3^ contained nucleophilic sites. Since it had already been shown that silyl protected intermediate **17** could be readily converted into **3** (Scheme 2), the silyl protected imine derivative **23** was chosen for this role (Scheme 3). It permitted alkylation to **24** without side reactions. This provided access to many additional derivatives **2** after deprotection of the silyl group (**25** to **26**), activation of the alcohol for displacement reactions (through either the mesylate **27a** or the more reactive iodide **27b**) and displacement with a variety of amines.

Model studies to **2** were performed with four alkylating agents R^1^X: [R^1^ = CH_2_Ph, CH_2_CH=CH_2_; (CH_2_)_7_CH_3_; CH_2_Ph-4-CF_2_-P(O)(OEt)_2_] (Scheme 3). The acylating agent (R^2^ = 4-NC-Ph), nucleophile R^3^ and chain length were kept constant. In addition these initial studies, which focused on the compatibility of the protected silyl alcohol with alkylation chemistry, utilized the alcohol activation and amine incorporation procedures (*via* mesylate **27a**) employed in the earlier lantern-based work [11]. Products for this 11-step synthesis were obtained in moderate overall yields (Table 1). Structure **2d**, which incorporates a stable, protected phosphotyrosine analog [6], gives a particular example of the interesting types of molecules available by this route.

**Table 1:** Survey of alkylating agents with incorporation of a single amine.

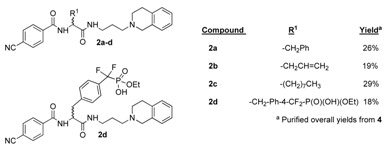

In this preliminary work a variety of by-products were formed during the alcohol to amine transformation (**26** to **28** *via* **27a**, Scheme 3). To minimize side-reactions and provide an alternative activation procedure, we developed a modified route through iodide intermediate **27b**. A direct comparison of these two routes gave **2a** in 65% crude purity via the mesylate intermediate **27a** and 88% crude purity via the iodide **27b**. We utilized this procedure (through the iodide) to create 32 new compounds and demonstrate the ability to introduce three points of variability (with basic functionality in R^3^) in structure **2**. The results with purified yields are shown in Table 2.

**Table 2:** 32 compounds from 4 alkylating agents, 4 carboxylic acids and 2 amines.

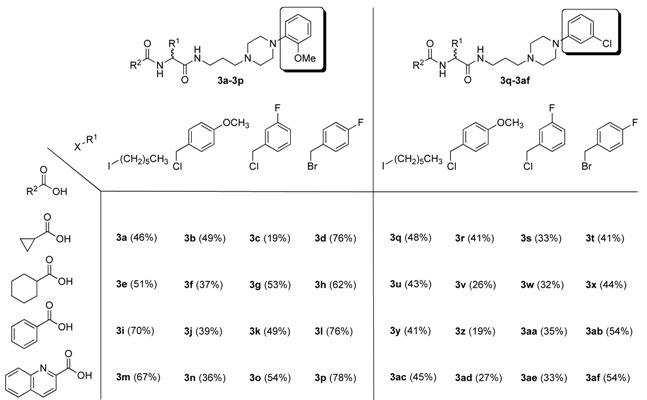

## Conclusions

This procedure provides ready access to the fundamental peptide scaffold **1** with multiple substitutions at positions R^1^, R^2^, and R^3^. The average overall purified yield to combinatorially prepare the 32 compounds **3a-3af** using this 11-step synthetic sequence was 46%. The R^3^ site can contain basic residues. Alternatively, based on ample precedent [12], the key iodide intermediate **27b** could provide access to many other interesting and valuable derivatives of **2** *via* simple nucleophilic displacement reactions.

## Experimental

### General

Organic solvents were of reagent grade and used directly without purification. Chloroform-*d*_1_, 1-chloro-3-iodopropane, 1-(3-chlorophenyl)piperazine, cyclohexanecarboxylic acid, DIPCDI (*N,N’*-di-isopropylcarbodiimide), anhydrous DMF (*N,N-*dimethylformamide), 1-iodohexane and methane-sulfonyl chloride were purchased from Acros Organics. TFA (trifluoroacetic acid) and hydrochloric acid were obtained from Fisher Scientific. BTPP [*tert*-butylimino-tri(pyrrolidino)phosphorane] was purchased from Fluka. 3-Amino-1-propanol, benzoic acid, benzoyl peroxide, NBS (*N*-bromo-succinimide), *tert*-butylchlorodiphenylsilane, 1-chloro-4-iodobutane, 1-chloro-5-iodopentane, cyclo-propanecarboxylic acid, 4-cyanobenzoic acid, 3,4-dichlorobenzaldehyde, 1-(2,3-dichlorophenyl)-piperazine hydrochloride, α,α’-dichloro-*o*-xylene, DAST [(diethylamino)sulfur trifluoride], DIEA (*N,N*-diisopropylethylamine), HOBt (1-hydroxybenzotriazole hydrate), imidazole, 1-iodooctane, methanol-*d*_4_, 1-(2-methoxyphenyl)piperazine, anhydrous NMP (1-methyl-2-pyrrolidinone), piperidine, anhydrous pyridine, quinaldic acid, 1,2,3,4-tetrahydroisoquinoline, TBAF (tetrabutylammonium fluoride), triethylamine, anhydrous TMOF (trimethyl orthoformate), and triphenylphosphine were obtained from Aldrich Chemical Co. Iodine was obtained from J. T. Baker. 4-Methylbenzhydrylamine hydrochloride resin (PL-MBHA·HCl, 1.6 mmol/g, 75–150 μm) was purchased from Polymer Laboratories. 4-(4-Formyl-3,5-dimethoxyphenoxy)butyric acid (BAL linker), Fmoc-Gly-OH (*N*-Fmoc-glycine), and HBTU (2-(1*H*-benzotriazole-1-yl)-1,1,3,3-tetramethylaminium hexafluorophosphate) were purchased from NovaBiochem. Diethyl [(4-α-bromomethyl)phenyldifluoromethyl]phosphonate [6] and *O*-*tert*-butyldiphenylsilyl-3-aminopropanol hydrochloride [13] were prepared according to literature procedures. Solution and solid-phase organic transformations and resin washes were carried out at ambient temperature, unless indicated otherwise. Ratios in all solvent and reagent mixtures prepared are volume to volume unless otherwise noted.

Manual solid-phase organic syntheses were carried out in two types of reaction vessels. Peptide synthesis reaction vessels (50 mL) with coarse porosity fritted glass support and supplied with a GL thread and a Teflon-lined PBT screw cap (ChemGlass, CG-1860-03) were used for large scale (up to 3.7 mmol) reactions. Small scale reactions (typically 50 μmol) were performed in 3.5 mL fritted glass reaction vessels (Chemglass, IUP-0305-270H) equipped with polypropylene screw caps (Chemglass, CV-3730-G013) with Teflon-faced silicon septa (Chemglass, CV-4080-0013) on the Bill-Board set, which was designed by one of us (WLS) as inexpensive equipment [2,14] to simplify and expedite multiple, manual solid-phase syntheses. For agitation purpose, the large scale reactions in the peptide synthesizers were placed on an orbital shaker Roto Mix (Type 50800 by Thermolyne) while appropriate motor rotators were used as rotation apparatus for small scale reactions.

Depending on the number of reactions to be performed, the starting resin was distributed either by weight or as aliquots from an isopycnic suspension [2]. In the case of distribution by volume from an isopycnic suspension, the Bill-Boards were placed in their drain trays, and from a neutral buoyancy suspension in CH_2_Cl_2_–NMP, 50 μmols of the starting resin (with a known loading) was typically distributed, *via* repeated aliquots (1 mL), to each of the reaction vessels in a given Bill-Board (6-pack or 24-pack). During the distribution of the resin, the isopycnic solvent was allowed to drain through the frit in the reaction vessels. When distribution was complete, residual solvent was removed with an “air-push” from a disposable plastic pipet (Fisher, 13-711-23) fitted with a pierced septum (Aldrich, Z 12743-4). The resin was then washed with an appropriate solvent (this solvent wash was also carried out when the resin was weighed into the reaction vessels). The bottom of each reaction vessel was then capped, and a new calibrated pipet (Fisher, 13-711-24) was used for adding each reagent in the following step. The tops of all reaction vessels were capped and the Bill-Board was placed on an appropriate rotation apparatus. Following the reaction the reagents and solvents were drained and the resin product was then washed with the indicated solvents. Resin-bound intermediates were air-dried after the final CH_2_Cl_2_ washes, unless re-weighing was necessary, in which case overnight drying was carried out under high vacuum (≤ 0.2 mm Hg) or under low vacuum (house vacuum) for 24–36 h in a vacuum desiccator. During resin washing with solvents for large scale reactions, at least 3 min of solvent contact with the resin in the reaction vessels (bottom closed) was performed, then the resin was drained, followed by an air-push. For washing of small scale reactions, at least 30 sec was normally used after addition of solvents to the reaction vessels (with bottom end open for draining) followed by an air-push. Solid-phase reactions at elevated temperatures (50 °C, 60 °C and/or 80 °C) were carried out in an Isotemp® Oven Model 280A (Fisher Scientific) with the reaction vessels capped to finger tightness.

Analytical thin layer chromatography (TLC) was performed with EM Science silica gel 60 F_254_, 0.25 mm pre-coated glass plates (EMD Chemical Inc., 5715-7). TLC plates were visualized using UV_254_. Column chromatography was performed on HyperSep SI® 3 mL cartridges (60108-315) pre-loaded with 500 mg of silica gel 60 (irregular particles 40–63 μm) from Thermo Electron Corporation. The yields of the final compounds, after chromatographic purification, were calculated on the basis of the initial loading of the starting resins and are the overall yields for all reaction steps starting from these resins. ^1^H-NMR (500 MHz) and ^13^C -NMR (125 MHz) spectra were recorded on a Bruker Avance III 500 spectrometer. Chemical shifts are reported in parts per million (ppm) and are referenced to the centerline of chloroform-*d*_1_ (δ 7.26 ppm ^1^H-NMR, 77.0 ppm ^13^C-NMR) using TMS (0.00 ppm), or chloroform-*d*_1_ mixed with methanol-*d*_4_ (2–10%). Coupling constants are given in Hertz (Hz).

Electrospray ionization mass spectrometry was conducted using a PESciex API III triple stage quadrupole mass spectrometer operated in either positive-ion or negative-ion detection mode. LC/MS analyses were conducted using an Agilent system, consisting of a 1100 series HPLC connected to a diode array detector and a 1946D mass spectrometer configured for positive-ion/negative-ion electro-spray ionization. The LC/MS samples were analyzed as solutions in CH_3_CN, prepared at 0.08–0.12 mg/mL concentration. The LC/MS derived composition of mixtures was determined based on UV integration at 210 nm. The LC/MS chromatography was carried out on an Agilent Zorbax SB–C_8_ column (PN835975-906; 4.6 × 50 mm, 3.5 μm) with linear gradients of 0.1% TFA in CH_3_CN and 0.1% aqueous TFA and were run at 1.0 mL/min flow rate from 20:80 to 90:10 for 25 min. The composition of reaction mixtures was determined based on the integration of NMR spectra as well as LC/MS results. High-resolution mass spectrometry was obtained on a MAT 95XP (Thermo Electron Corp.) with chemical ionization (CI), electron impact (EI), or fast-atom bombardment (FAB) mode.

### General procedures for manual solid-phase organic syntheses

*Preparation of MBHA-BAL Resin* **4**: PL-MBHA·HCl resin (1.03 g, 1.65 mmol, 1.60 mmol/g) loaded in a 50 mL peptide synthesis reaction vessel was sequentially washed with DMF (3 × 17 mL), CH_2_Cl_2_ (2 × 17 mL), 10% DIEA/CH_2_Cl_2_ (5 × 17 mL) and CH_2_Cl_2_ (2 × 17 mL). A solution of 4-(4-formyl-3,5-dimethoxyphenoxy)butyric acid (1.77 g, 6.60 mmol, 4 equiv), HBTU (2.50 g, 6.60 mmol, 4 equiv) and DIEA (2.30 mL, 13.2 mmol, 8 equiv) in anhydrous DMF (23 mL) prepared immediately prior to the reaction was then added to the washed resin. The reaction mixture was agitated on an orbital shaker for 18 h. The completion of the reaction was confirmed by a negative chloranil test [15]. The resultant orange yellow MBHA-BAL resin **4** was washed with DMF (3 × 17 mL) and CH_2_Cl_2_ (4 × 17 mL), and dried under low vacuum for 24 h.

*Preparation of the Secondary Amine-Bound Resin* **21** *by Reductive Amination:* The dried MBHA-BAL resin **4** (1.51 g, 1.65 mmol) was swelled in CH_2_Cl_2_ (17 mL) for 1 h, followed by DMF washes (3 × 17 mL). *O*-*tert*-Butyldiphenylsilyl-3-aminopropanol hydrochloride [13] (8.66 g, 24.75 mmol, 15 equiv) was added as a solid followed by addition of DMF–TMOF (1:2, 28 mL) to the resin. Theresulting reaction mixture was heated to 60 °C for 24 h. The resin-bound Schiff base product was washed with DMF (4 × 17 mL), THF (5 × 17 mL), MeOH (2 × 17 mL), CH_2_Cl_2_ (2 × 17 mL), and MeOH (3 × 17 mL). After the resulting imine resin was swelled in CH_2_Cl_2_ (17 mL) for 30 min followed by THF (17 mL) wash, it was treated with a solution of NaBH_3_CN (1.04 g, 16.5 mmol, 10 equiv) in 5% HOAc in THF–MeOH (1:1, 28 mL). The reaction mixture was agitated on an orbital shaker for 18 h. The resin was drained and washed with THF–MeOH (1:1, 3 × 17 mL), MeOH (2 × 17 mL), THF (3 × 17 mL) and CH_2_Cl_2_ (3 × 17 mL). The amine resin product **21** was air-dried and was then used directly for subsequent coupling reactions.

*Coupling of the Amine Resin* **21** *with N-Fmoc-Glycine using HBTU to Prepare Resin-Bound* **22***:* The amine resin **21** (1.65 mmol) was swelled in CH_2_Cl_2_ (17 mL) for 30 min, and then it was washed with CH_2_Cl_2_–DMF (85:15, 17 mL). Fmoc-Gly-OH (2.45 g, 8.25 mmol, 5 equiv), HBTU (3.13 g, 8.25 mmol, 5 equiv), CH_2_Cl_2_–DMF (85:15, 39 mL) and DIEA (2.87 mL, 16.5 mmol, 10 equiv) were then added to the reaction vessel. The reaction mixture was agitated on an orbital shaker for 42 h. The completion of the reaction was indicated by a negative chloranil test [15]. The resulting Fmoc-Gly-BAL resin product **22** was then washed with DMF (3 × 17 mL), THF–MeOH (1:1, 3 × 17 mL), THF (3 × 17 mL), and CH_2_Cl_2_ (3 × 17 mL). The washed resin **22** was dried under low vacuum for 24–36 h.

*Preparation of the 3,4-Dichlorobenzaldehyde Imine of Glycine on BAL Resin* **23**: The resin **22** (1.65 mmol) was swelled in CH_2_Cl_2_ (17 mL) for 30 min, and washed with DMF (5 × 17 mL), then it was treated with 20% piperidine/DMF (23 mL, 20 min) and subsequently washed with DMF (6 × 17 mL). The resin was swelled in CH_2_Cl_2_ (17 mL) for 30 min and washed with NMP (4 × 17 mL). A solution of 3,4-dichlorobenzaldehyde (1.25 M, 19.8 mL, 24.75 mmol, 15 equiv) in NMP–TMOF (1:2) was then added to the reaction vessel and the reaction mixture was rotated for 24 h. The resulting resin-bound Schiff base product **23** was sequentially washed with NMP (4 × 17 mL), THF (3 × 17 mL), CH_2_Cl_2_ (3 × 17 mL) and dried under low vacuum for 24 h.

*Alkylation of the Aldimine of Glycine on BAL Resin* **23** *with Alkyl Halides, Subsequent Imine Hydrolysis, and Acylation with Carboxylic Acids:* After resin-bound Schiff base **23** (50 μmol) was pre-swelled in CH_2_Cl_2_ for 1 h, it was washed with NMP (4 × 1 mL). To the resin was then added a solution of R^1^X in NMP (2.0 M, 0.25 mL, 0.5 mmol, 10 equiv), followed by addition of BTPP solution in NMP (2.0 M, 0.25 mL, 0.5 mmol, 10 equiv). Alkylation was allowed to proceed at ambient temperature for 24 h with rotation. The alkylated resin product was filtered and washed with NMP (4 × 1 mL), CH_2_Cl_2_ (4 × 1 mL) and THF (3 × 1 mL). The resin was then treated with 1N HCl–THF (1:2, 1 mL) for 20 min. The resulting resin was filtered and washed with THF (3 × 1 mL), 10% DIEA/CH_2_Cl_2_ (5 × 1 mL), CH_2_Cl_2_ (2 × 1 mL). After the resin was swelled in CH_2_Cl_2_ (1 mL) for 1 h, and washed with DMF (3 × 1 mL), to the resin was added a solution of carboxylic acid R^2^COOH and HOBt in DMF (0.38 M, 0.66 mL, 0.25 mmol, 5 equiv), which was pre-mixed (6 - 10 min before addition) with DIPCDI (neat, 32 mg, 0.25 mmol, 5 equiv). The reaction mixture was allowed to rotate for 18 h. The completion of the reaction was confirmed by a negative chloranil test [15], and the filtered resin product **25** was washed with DMF (4 × 1 mL), THF (3 × 1 mL), CH_2_Cl_2_ (3 × 1 mL), and dried in air.

*Model Studies for the Synthesis of* **2a**–**2d** *via Deprotection of Silyl Ether* **25** *with TBAF, Subsequent Mesylation of Alcohol* **26** *using Mesyl Chloride in Pyridine, and N-Alkylation with Tetrahydroisoquinoline:* The acylated resin product **25** (50 μmol) was washed with THF (3 × 1 mL), and swelled in THF for 1 h. TBAF (1 M in THF, 1.0 mL, 1.0 mmol, 20 equiv) was added to the drained resin, and the reaction mixture was allowed to rotate for 18 h. The drained resin was then washed with THF (5 × 1 mL) and CH_2_Cl_2_ (3 × 1 mL). After the alcohol resin **26** was swelled in CH_2_Cl_2_ for 1 h, a suspension of anhydrous pyridine with MsCl (1 M, 1.0 mL, 0.5 mmol, 20 equiv) was added to the resin, and the resulting mixture was rotated for 1 h. The drained resin product **27a** was washed with DMF (3 × 1 mL), H_2_O (1 × 1 mL), DMF (2 × 1 mL) and CH_2_Cl_2_ (5 × 1 mL). After the air-dried resin was swelled in CH_2_Cl_2_ for 30 min, to the resin was added a solution of tetrahydroisoquinoline in DMSO (1 M, 0.75 mL, 0.75 mmol, 15 equiv), and the reaction mixture was heated to 80 °C for 5 h. The resulting resin product was drained, washed with DMF (2 × 1 mL), H_2_O (2 × 1 mL), DMF (3 × 1 mL), CH_2_Cl_2_ (4 × 1 mL), and air-dried. The resin product was then cleaved with 50% TFA/CH_2_Cl_2_ (1 mL) over 1.5 h, and the filtrate of the reaction mixture was collected and combined with washes of CH_2_Cl_2_ (2 × 1 mL) of the resin. A 100 μL sample of the combined solution was analyzed for crude purity by LC/MS. The cleavage solution was evaporated with a stream of nitrogen in a contained system with trapping of the evaporated TFA in 2N NaOH. The crude residue was re-dissolved in CH_2_Cl_2_, or CH_2_Cl_2_ with MeOH, if needed (total solution volume ≤0.5 mL), and purified using a pre-loaded silica gel cartridge with CH_2_Cl_2_–MeOH (95:5 or 93:7) to elute the purified product. Following solvent removal under N_2_ flow, the purified product **2a–2d** was normally obtained as an amorphous white solid or light yellow oil.

*α-[(4-Cyanobenzoyl)amino]-N-[(3,4-dihydroisoquinolin-2(1H)-yl)propyl]-benzenepropanamide* (**2a**): Product **2a** was obtained from resin **23**, benzyl bromide, 4-cyanobenzoic acid, and tetrahydroisoquinoline as an amorphous white solid (6 mg, 26% isolated yield) following chromatographic purification over silica gel with CH_2_Cl_2_–MeOH (93:7). Initial LC/MS purity 65%, *t*_R_ = 7.3 min; ^1^H-NMR (CD_3_OD/CDCl_3_): δ 1.67–1.78 (m, 2H), 2.49–2.61 (m, 3H), 2.74–2.86 (m, 2H), 2.88–2.93 (m, 1H), 2.96 (dd, *J* = 8.0 Hz, *J* = 13.5 Hz, 1H), 3.03 (dd, *J* = 6.0 Hz, *J* = 13.5 Hz, 1H), 3.24–3.32 (m, 1H), 3.37–3.44 (m, 1H), 3.51–3.61 (m, 2H), 4.53 (dd, *J* = 7.7 Hz, *J* = 13.8 Hz, 1H), 6.95–7.01 (m, 2H), 7.02–7.06 (m, 2H), 7.12–7.18 (m, 3H), 7.19–7.23 (m, 3H), 7.32–7.36 (m, 1H), 7.69 (d, *J* = 8.5 Hz, 2H), 7.79 (d, *J* = 8.5 Hz, 2H). ^13^C-NMR (CD_3_OD/CDCl_3_): δ 23.7, 24.2, 35.8, 37.8, 49.1, 51.9, 52.5, 55.8, 115.2, 117.9, 126.1, 126.8, 126.9, 127.6, 128.1, 128.6, 128.7, 128.9, 129.2, 130.1, 132.2, 136.6, 137.3, 166.2, 172.6. HRMS calcd. for (M + H)^+^: C_29_H_31_N_4_O_2_ 467.2447, found 467.2431.

*2-[(4-Cyanobenzoyl)amino]-N-[(3,4-dihydroisoquinolin-2(1H)-yl)propyl]-4-pentenamide* (**2b**): This product was obtained from resin **23**, allyl bromide, 4-cyanobenzoic acid, and tetrahydroisoquinoline as a light yellow oil (4 mg, 19% isolated yield) following chromatographic purification over silica gel with CH_2_Cl_2_–MeOH (92:8). Initial LC/MS purity 60%, *t*_R_ = 5.4 min; ^1^H-NMR (CD_3_OD/CDCl_3_): δ 1.97–2.04 (m, 2H), 2.49–2.58 (m, 1H), 2.60–2.68 (m, 1H), 3.06–3.11 (m, 3H), 3.24–3.31 (m, 1H), 3.32–3.38 (m, 1H), 3.40–3.48 (m, 2H), 4.03–4.19 (m, 2H), 4.29 (s, 1H), 4.61 (dd, *J* = 6.0 Hz, *J* = 7.7 Hz, 1H), 5.05–5.21 (m, 2H), 5.65–5.82 (m, 1H), 7.06 (d, *J* = 7.5 Hz, 1H), 7.08–7.14 (m, 1H), 7.18 (d, *J* = 7.1 Hz, 1H), 7.21–7.28 (m, 3H), 7.70 (d, *J* = 8.3 Hz, 2H), 7.97 (d, *J* = 8.5 Hz, 2H). ^13^C-NMR (CD_3_OD/CDCl_3_): δ 24.2, 25.3, 36.4, 36.7, 41.5, 44.4, 53.5, 53.8, 115.3, 118.0, 118.9, 126.6, 126.7, 127.2, 127.3, 128.1, 128.9, 129.0, 132.3, 132.8, 137.4, 166.1, 172.3. HRMS calcd. for (M + H)^+^: C_25_H_29_N_4_O_2_ 417.2212, found 417.2200.

*2-[(4-Cyanobenzoyl)amino]-N-[(3,4-dihydroisoquinolin-2(1H)-yl)propyl]decanamide* (**2c**): Product **2c** was obtained from resin **23**, 1-iodooctane, 4-cyanobenzoic acid, and tetrahydroisoquinoline as a light yellow oil (7 mg, 29% isolated yield) following chromatographic purification over silica gel with CH_2_Cl_2_–MeOH (95:5). Initial LC/MS purity 65%, *t*_R_ = 11.8 min; ^1^H-NMR (CD_3_OD/CDCl_3_): δ 0.86 (t, *J* = 7.0 Hz, 3H), 1.17–1.48 (m, 12H), 1.71–1.95 (m, 2H), 1.99–21.6 (m, 2H), 2.99–3.52 (m, 7H), 3.55–3.79 (m, 1H), 3.92–4.25 (m, 1H), 4.30–4.69 (m, 2H), 7.03-7.15 (m, 1H), 7.20 (d, *J* = 7.1 Hz, 1H), 7.24–7.34 (m, 3H), 7.71 (d, *J* = 8.5 Hz, 2H), 8.00 (d, *J* = 8.7 Hz, 2H). ^13^C-NMR (CD_3_OD/CDCl_3_): δ 14.0, 22.6, 23.8, 24.3, 25.9, 29.1, 29.2, 29.3, 31.7, 32.0, 35.7, 49.3, 52.2, 52.6, 55.0, 115.2, 118.0, 126.1, 126.8, 127.6, 128.2, 128.7, 128.9, 130.1, 132.2, 137.4, 166.5, 171.1. HRMS calcd. for (M + H)^+^: C_30_H_41_N_4_O_2_ 489.3151, found 489.3138.

*α -[(4-Cyanobenzoyl)amino]-4-[(ethoxyhydroxyphosphinyl)difluoromethyl]-N-[(3,4-dihydroisoquinolin-2(1H)-yl)propyl]benzenepropanamide* (**2d**): Product **2d** was obtained from resin **23**, diethyl [(4-α-bromomethyl)phenyldifluoromethyl]-phosphonate, 4-cyanobenzoic acid, and tetrahydroisoquinoline as a light yellow oil (6 mg, 19% isolated yield) following chromatographic purification over silica gel with CH_2_Cl_2_–MeOH (92:8). Initial LC/MS purity 47%, *t*_R_ = 5.9 min; ^1^H-NMR (CD_3_OD/CDCl_3_): δ 1.05–1.19 (m, 3H), 1.30–1.41 (m, 2H), 2.57–2.80 (m, 3H), 3.03–3.43 (m, 10H), 4.04–4.16 (1H), 4.25 (s, 1H), 4.70–4.83 (m, 1H), 7.08–7.36 (m, 8H), 7.56 (d, *J* = 7.3 Hz, 2H), 7.79 (d, *J* = 8.0 Hz, 2H), 8.01 (d, *J* = 8.2 Hz, 2H). ^13^C-NMR (125 MHz, CD_3_OD/CDCl_3_): δ 16.3, 22.9, 24.7, 36.1, 38.9, 41.6, 52.6, 52.8, 55.5, 62.6, 115.1, 117.9, 126.5, 126.7, 126.9, 127.1, 127.7, 128.0, 128.2, 128.5, 128.7, 128.9, 130.5, 132.2, 137.7, 165.6, 170.8. HRMS calcd. for (M + H)^+^: C_32_H_37_F_2_N_4_O_5_P 625.2313, found 625.2326.

*Direct Comparison of Activation of Alcohol Resin **26** via Mesylation (to* **27a***) with that via Iodination (to* **27b***) using Optimal N-Alkylation and Cleavage Conditions:* The mesylation of the alcohol resin **26** was the same as described above. Iodination of the resin **26** was performed as follows. After the free alcohol resin **26** was swelled in CH_2_Cl_2_ for 1 h and washed with DMF (3 × 1 mL), a pre-mixed solution of iodine (63 mg, 0.25 mmol, 5 equiv), PPh_3_ (66 mg, 0.25 mmol, 5 equiv), and imidazole (17 mg, 0.25 mmol, 5 equiv) in DMF (1.0 mL) was added. After 18 h, the filtered resin was washed with DMF (3 × 1 mL), MeOH (3 × 1 mL), DMF (2 × 1 mL), and CH_2_Cl_2_ (3 × 1 mL). After mesylated resin **27a** and iodinated resin **27b** were swelled in CH_2_Cl_2_ for 40 min, to each resin was added a solution of tetrahydroisoquinoline in DMSO (1 M, 0.75 mL, 0.75 mmol, 15 equiv). The reaction was heated to 80 °C over 3 h for mesylated resin or 50 °C over 6 h for iodinated resin. The resulting resin products were drained, sequentially washed with DMF (2 × 1 mL), MeOH (2 × 1 mL), DMF (3 × 1 mL), CH_2_Cl_2_ (4 × 1 mL), and cleaved with 90% TFA/CH_2_Cl_2_ (1 mL) over 1.5 h. The filtrate of the reaction mixture was collected and combined with washes of 50% TFA/CH_2_Cl_2_ (1 × 1 mL) and CH_2_Cl_2_ (1 × 1 mL) of the resin. A 100 μL sample of the combined solution was analyzed for crude purity by LC/MS. The crude purity for the product **2** was found to be 65% for the product through the mesylation and 88% for the product through the iodination process.

*Synthesis of 32 New Resin Products* **25** *(Targeting Piperazine Derivatives* **3** *) from the Same Aldimine of Glycine on BAL Resin* **23** *through Alkylation, Hydrolysis, and Acylation:* Resin-bound Schiff base **23** (1.65 mmol) pre-swelled in CH_2_Cl_2_ for 1 h was evenly distributed to 33 of the reaction vessels in two separate 24-pack BillBoards *via* an isopycnic solution in CH_2_Cl_2_–NMP (9:5, v/v). 32 of the reaction vessels were arranged as two 4 × 4 grids on the BillBoards, and the 33^rd^ reaction vessel was put at position on A5 on one of the BillBoards for the quality control experiment for the resin **23**. After the drained resin (50 μmol) was washed with NMP (4 × 1 mL), to the four reaction vessels down the first column positions (i.e. A1, B1, C1 and D1) on both BillBoards were added 1-iodohexane (2.0 M, 0.25 mL, 0.50 mmol, 10 equiv). To the four reaction vessels down the 2^nd^ column positions on both BillBoards were added 4-methoxybenzyl chloride (2.0 M, 0.25 mL, 0.50 mmol, 10 equiv). To the four reaction vessels down the 3^rd^ column positions on both BillBoards were added 3-fluorobenzyl chloride (2.0 M, 0.25 mL, 0.50 mmol, 10 equiv). To the four reaction vessels down the 4^th^ column positions on both BillBoards were added 4-fluorobenzyl bromide (2.0 M, 0.25 mL, 0.50 mmol, 10 equiv). To the control reaction vessel was added a solution of BnBr in NMP (2.0 M, 0.25 mL, 0.50 mmol, 10 equiv) for quality control of the starting resin **23**.

Then a solution of BTPP in NMP (2.0 M, 0.25 mL, 0.50 mmol, 10 equiv) was added to each of the 33 reaction vessels. Alkylation was allowed to proceed at ambient temperature for 24 h with rotation. The alkylated resin product was filtered and washed with NMP (4 × 1 mL), CH_2_Cl_2_ (4 × 1 mL) and THF (3 × 1 mL). The resin was then treated with 1N HCl–THF (1:2, 1 mL) for 20 min. The resulting resin was filtered and washed with THF (3 × 1 mL), 10% DIEA/CH_2_Cl_2_ (5 × 1 mL), CH_2_Cl_2_ (2 × 1 mL). After the resin was swelled in CH_2_Cl_2_ (1 mL) for 1 h, and washed with DMF (3 × 1 mL), to the resins across row A positions (i.e. A1, A2, A3, A4) on both BillBoards were added a solution of cyclopropanecarboxylic acid and HOBt in DMF (0.38 M, 0.66 mL, 0.25 mmol, 5 equiv) which was pre-mixed (6 - 10 min before addition) with DIPCDI (32 mg, 0.25 mmol, 5 equiv). To the resins across row B positions on both BillBoards were added a solution of cyclohexanecarboxylic acid and HOBt in DMF (0.38 M, 0.66 mL, 0.25 mmol, 5 equiv) which was pre-mixed (6 - 10 min before addition) with DIPCDI (32 mg, 0.25 mmol, 5 equiv). To the resins across row C positions on both BillBoards were added a solution of benzoic acid and HOBt in DMF (0.38 M, 0.66 mL, 0.25 mmol, 5 equiv) which was pre-mixed (6 - 10 min before addition) with DIPCDI (32 mg, 0.25 mmol, 5 equiv). To the resins across row D positions on both BillBoards were added a solution of quinaldic acid and HOBt in DMF (0.38 M, 0.66 mL, 0.25 mmol, 5 equiv) which was pre-mixed (6 min before addition) with DIPCDI (32 mg, 0.25 mmol, 5 equiv; and it was observed that the colorless clear solution turned purple 2 min after DIPCDI was added). To the quality control reaction vial was added a solution of benzoic acid and HOBt in DMF (0.38 M, 0.66 mL, 0.25 mmol, 5 equiv) which was pre-mixed (6 - 10 min before addition) with DIPCDI (neat, 32 mg, 0.25 mmol, 5 equiv). The reaction mixture was rotated for 18 h. The completion of the reaction was confirmed by a negative chloranil test [15], and the filtered resin product **25** was washed with DMF (4 × 1 mL), THF (3 × 1 mL), CH_2_Cl_2_ (3 × 1 mL), and dried in air.

*Deprotection of the Silyl Ether on Resin* **25** *with TBAF and Subsequent Iodination using Triphenylphosphine, Iodine and Imidazole to Form Resin-Bound* **27b**: The resin product **25** was swelled in CH_2_Cl_2_ for 40 min, and the drained resin was washed with THF (3 × 1 mL). To the resin was added TBAF/THF (1 M, 1.0 mL, 1.0 mmol, 20 equiv), and the reaction was allowed to proceed for 18 h. The drained resin was washed with THF (3 × 1 mL), and CH_2_Cl_2_ (3 × 1 mL), and air dried. After this alcohol resin was swelled in CH_2_Cl_2_ for 1 h and washed with DMF (3 × 1 mL), a pre-mixed solution of iodine (63 mg, 0.25 mmol, 5 equiv), PPh_3_ (66 mg, 0.25 mmol, 5 equiv), and imidazole (17 mg, 0.25 mmol, 5 equiv) in DMF (1.0 mL) was added. After 18 h, the filtered resin was washed with DMF (3 × 1 mL), MeOH (3 × 1 mL), DMF (2 × 1 mL), and CH_2_Cl_2_ (3 × 1 mL).

*Displacement of Iodo-Resin* **27b** *with Amines and Subsequent Cleavage to Target Molecules* **3**: The air-dried resin **27b** was swelled in CH_2_Cl_2_ for 40 min, and to the first 16 reaction vessels on one BillBoard were added a solution of 2-methoxyphenyl piperazine in anhydrous DMSO (1 M, 0.75 mL, 0.75 mmol, 15 equiv), while to the other 16 reaction vessels on the other BillBoard were added a solution of 3-chlorophenyl piperazine in anhydrous DMSO (1 M, 0.75 mL, 0.75 mmol, 15 equiv), and to the resin for quality control was added a tetrahydroisoquinoline solution in DMSO (1M, 0.75 mL, 0.75 mmol, 15 equiv). All reaction vessels were heated to 50 ◦C for 6 h with occasional agitation. The resulting resin product was drained, sequentially washed with DMF (2 × 1 mL), MeOH (2 × 1 mL), DMF (3 × 1 mL), CH_2_Cl_2_ (4 × 1 mL), and cleaved with 90% TFA/CH_2_Cl_2_ (1 mL) over 1.5 h, affording the target compound **3**.

*2-[(Cyclopropanecarbonyl)amino]-N-{[4-(2-methoxyphenyl)-piperazin-1-yl]propyl}-octanamide* (**3a**): Product **3a** was obtained from resin **23**, 1-iodohexane, cyclopropanecarboxylic acid, and 2-methoxy-phenylpiperazine as an amorphous light yellow solid (10.5 mg, 46% isolated yield) following chromatographic purification over silica gel with CH_2_Cl_2_–MeOH (92:8). Initial LC/MS purity 81%, *t*_R_ = 8.2 min; ^1^H-NMR (CDCl_3_): δ 0.71–0.78 (m, 2H), 0.86 (t, *J* = 6.9 Hz, 3H), 0.89–0.96 (m, 2H), 1.19–1.38 (m, 10H), 1.42–1.51 (m, 1H), 1.57–1.69 (m, 1H), 1.79–1.90 (m, 3H), 2.74–2.83 (m, 2H), 2.89–3.02 (m, 3H), 3.18–3.28 (m, 3H), 3.33–3.43 (m, 2H), 3.87 (s, 3H), 4.36 (dd, *J* = 7.8 Hz, *J* = 13.7 Hz, 1H), 6.60 (d, *J* = 7.1 Hz, 1H), 6.88 (d, *J* = 7.7 Hz, 1H), 6.91–6.97 (m, 2H), 7.01–7.09 (m, 2H), 7.52–7.65 (m, 1H). ^13^C-NMR (CDCl_3_): δ 7.3, 7.4, 14.0, 14.6, 22.6, 24.4, 25.7, 29.0, 31.7, 32.8, 37.7, 49.3, 53.0, 53.8, 55.4, 55.9, 111.3, 118.6, 121.2, 123.8, 140.1, 152.2, 172.7, 173.8. HRMS calcd. for (M + H)^+^: C_26_H_42_N_4_O_3_ 459.3335, found 459.3313.

*2-[(Cyclopropanecarbonyl)amino]-N-{[4-(2-methoxyphenyl)-piperazin-1-yl]propyl}-3-(4-methoxy-phenyl)propanamide* (**3b**): Product **3b** was obtained from resin **23**, 4-methoxybenzyl chloride, cyclopropanecarboxylic acid, and 2-methoxyphenylpiperazine as light yellow oil (12.2 mg, 49% isolated yield) following chromatographic purification over silica gel with CH_2_Cl_2_–MeOH (93:7). Initial LC/MS purity 78%, *t*_R_ = 6.1 min; ^1^H-NMR (CDCl_3_): δ 0.67–0.77 (m, 2H), 0.84–0.95 (m, 2H), 1.43–1.53 (m, 1H), 1.78–1.90 (m, 2H), 1.97–2.07 (m, 1H), 2.37 (t, *J* = 8.1 Hz, 1H), 2.68–2.79 (m, 2H), 2.84 (s, 1H), 2.98–3.05 (m, 2H), 3.20–3.29 (m, 4H), 3.33–3.42 (m, 2H), 3.76 (s, 3H), 3.86 (s, 3H), 4.50–4.79 (m, 1H), 6.81 (d, *J* = 8.6 Hz, 2H), 6.86 (d, *J* = 8.5 Hz, 2H), 6.91–6.95 (m, 2H), 7.03–7.08 (m, 1H), 7.14 (d, *J* = 8.5 Hz, 2H), 7.46 (brs, 1H). ^13^C-NMR (CDCl_3_): δ 7.4, 7.5, 14.6, 17.7, 23.9, 29.6, 30.7, 37.6, 44.0, 48.7, 49.5, 52.8, 55.2, 55.5, 111.3, 113.9, 118.7, 121.2, 123.9, 128.8, 130.5, 139.7, 152.2, 158.5, 171.9, 174.0. HRMS calcd. for (M + H)^+^: C_28_H_39_N_4_O_4_ 495.2907, found 495.2914.

*2-[(Cyclopropanecarbonyl)amino]-N-{[4-(2-methoxyphenyl)-piperazin-1-yl]propyl}-3-(3-fluoro-phenyl)propanamide* (**3c**): Product **3c** was obtained from resin **23**, 3-fluorobenzyl chloride, cyclopropanecarboxylic acid, and 2-methoxyphenylpiperazine as light yellow oil (4.6 mg, 19% isolated yield) following chromatographic purification over silica gel with CH_2_Cl_2_–MeOH (93:7). Initial LC/MS purity 65%, *t*_R_ = 6.5 min; ^1^H-NMR (CDCl_3_): δ 0.66–0.82 (m, 2H), 0.85–0.99 (m, 2H), 1.32–1.49 (m, 1H), 1.61–1.81 (m, 2H), 2.48–2.64 (m, 2H), 2.65–2.87 (m, 3H), 3.03–3.22 (m, 5H), 3.24–3.47 (m, 3H), 3.87 (s, 3H), 4.49–4.69 (m, 1H), 6.53 (brs, 1H), 6.87 (d, *J* = 8.1 Hz, 1H), 6.90–6.97 (m, 4H), 6.98–7.06 (m, 2H), 7.21–7.25 (m, 1H), 7.34 (brs, 1H). ^13^C-NMR (CDCl_3_): δ 7.4, 7.5, 14.7, 24.4, 31.8, 38.7, 50.1, 53.2, 53.3, 54.9, 55.4, 77.6, 111.2 113.7, 116.4, 118.4, 121.1, 123.4, 125.1, 129.9, 139.4, 140.6, 152.2, 161.8, 163.8, 170.7, 173.5. HRMS calcd. for (M + H)^+^: C_27_H_36_FN_4_O_3_ 483.2771, found 483.2766.

*2-[(Cyclopropanecarbonyl)amino]-N-{[4-(2-methoxyphenyl)-piperazin-1-yl]propyl}-3-(4-fluoro-phenyl)propanamide* (**3d**): Product **3d** was obtained from resin **23**, 4-fluorobenzyl bromide, cyclopropanecarboxylic acid, and 2-methoxyphenylpiperazine as light yellow oil (18.3 mg, 76% isolated yield) following chromatographic purification over silica gel with CH_2_Cl_2_–MeOH (93:7). Initial LC/MS purity 83%, *t*_R_ = 6.4 min; ^1^H-NMR (CDCl_3_): δ 0.66–0.77 (m, 2H), 0.78–0.94 (m, 2H), 1.42–1.55 (m, 1H), 1.81–1.99 (m, 2H), 2.79–2.93 (m, 2H), 3.02 (dd, *J* = 7.4 Hz, *J* = 13.8 Hz, 2H), 3.09 (dd, *J* = 6.5 Hz, *J* = 13.8 Hz, 2H), 3.22–3.43 (m, 6H), 3.87 (s, 3H), 4.68 (dd, *J* = 7.3 Hz, *J* = 14.6 Hz, 1H), 6.89 (d, *J* = 8.4 Hz, 1H), 6.91–7.02 (m, 5H), 7.04–7.09 (m, 1H), 7.16–7.24 (m, 2H), 7.62 (brs, 1H). ^13^C-NMR (CDCl_3_): δ 7.4, 7.5, 14.7, 23.8, 36.5, 37.5, 48.1, 52.6, 54.8, 55.0, 55.4, 111.4, 115.2 115.4, 118.7, 121.2, 124.3, 130.9, 132.6, 139.2, 152.2, 160.9, 162.8, 172.1, 174.2. HRMS calcd. for (M + H)^+^: C_27_H_36_FN_4_O_3_ 483.2771, found 483.2755.

*2-[(Cyclohexanecarbonyl)amino]-N-{[4-(2-methoxyphenyl)-piperazin-1-yl]propyl}octanamide* (**3e**): Product **3e** was obtained from resin **23**, 1-iodohexane, cyclohexanecarboxylic acid, and 2-methoxy-phenylpiperazine as light yellow oil (12.8 mg, 51% isolated yield) following chromatographic purification over silica gel with CH_2_Cl_2_–MeOH (93:7). Initial LC/MS purity 81%, *t*_R_ = 10.8 min; ^1^H- NMR (CDCl_3_): δ 0.85 (t, *J* = 6.8 Hz, 3H), 1.20–1.30 (m, 11H), 1.36–1.48 (m, 2H), 1.58–1.68 (m, 2H), 1.73–1.88 (m, 7H), 1.97–2.07 (m, 0.5H), 2.08–2.21 (m, 1H), 2.38 (t, *J* = 8.1 Hz, 0.5H), 2.65–2.79 (m, 2H), 2.82–2.96 (m, 3H), 3.13–3.26 (m, 3H), 3.29–3.48 (m, 3H), 3.88 (s, 3H), 4.35 (dd, *J* = 7.6 Hz, *J* = 13.9 Hz, 1H), 6.38 (brs, 1H), 6.87 (d, *J* = 8.1 Hz, 1H), 6.89–6.96 (m, 2H), 6.99–7.06 (m, 1H), 7.64 (brs, 1H). ^13^C-NMR (CDCl_3_): δ 14.0, 17.7, 22.5, 24.6, 25.6, 25.7, 29.0, 29.3, 29.8, 30.7, 31.6, 32.8, 38.2, 45.3, 49.7, 53.2, 55.4, 111.3, 118.5, 121.1, 123.5, 140.4, 152.2, 172.5, 176.5. HRMS calcd. for (M + H)^+^: C_29_H_49_N_4_O_3_ 501.3805, found 501.3811.

*2-[(Cyclohexanecarbonyl)amino]-N-{[4-(2-methoxyphenyl)-piperazin-1-yl]propyl}-3(4-methoxy-phenyl)propanamide* (**3f**): Product **3f** was obtained from resin **23**, 4-methoxybenzyl chloride, cyclo-hexanecarboxylic acid, and 2-methoxyphenylpiperazine as light yellow oil (9.9 mg, 37% isolated yield) following chromatographic purification over silica gel with CH_2_Cl_2_–MeOH (93:7). Initial LC/MS purity 65%, *t*_R_ = 8.7 min; ^1^H-NMR (CDCl_3_): δ 1.16–1.27 (m, 3H), 1.32–1.43 (m, 2H), 1.61–1.69 (m, 3H), 1.72–1.83 (m, 4H), 1.97–2.04 (m, 1H), 2.05–2.13 (m, 1H), 2.38 (t, *J* = 8.1 Hz, 1H), 2.45–2.52 (m, 2H), 2.62–2.66 (m, 1H), 2.68–2.73 (m, 1H), 2.84 (s, 1H), 2.97 (d, *J* = 7.1 Hz, 2H), 3.06–3.13 (m, 2H), 3.22–3.34 (m, 2H), 3.38 (t, *J* = 7.1 Hz, 1H), 3.76 (s, 3H), 3.86 (s, 3H), 4.50 (dd, *J* = 7.2 Hz, *J* = 14.6 Hz, 1H), 6.30 (d, *J* = 7.2 Hz, 1H), 6.80 (d, *J* = 8.6 Hz, 2H), 6.87 (d, *J* = 7.9 Hz, 1H), 6.94 (d, *J* = 4.2 Hz, 2H), 6.99–7.05 (m, 1H), 7.11 (d, *J* = 8.6 Hz, 2H), 7.19 (brs, 1H). ^13^C-NMR (CDCl_3_): δ 17.7, 25.6, 25.7, 29.3, 29.7, 30.7, 38.1, 45.2, 49.5, 53.2, 54.7, 55.2, 55.4, 56.6, 111.2, 114.1, 118.4, 121.1, 123.3, 128.8, 130.4, 140.7, 152.2, 158.5, 171.1, 175.9. HRMS calcd. for (M + H)^+^: C_31_H_45_N_4_O_4_ 537.3441, found 537.3439.

*2-[(Cyclohexanecarbonyl)amino]-N-{[4-(2-methoxyphenyl)-piperazin-1-yl]propyl}-3-(3-fluoro-phenyl)propanamide* (**3g**): Product **3g** was obtained from resin **23**, 3-fluorobenzyl chloride, cyclo-hexanecarboxylic acid, and 2-methoxyphenylpiperazine as yellow oil (13.9 mg, 53% isolated yield) following chromatographic purification over silica gel with CH_2_Cl_2_–MeOH (93:7). Initial LC/MS purity 65%, *t*_R_ = 9.1 min; ^1^H-NMR (CDCl_3_): δ 1.14–1.37 (m, 5H), 1.60–1.78 (m, 5H), 1.93–2.07 (m, 3H), 2.08-2.18 (m, 1H), 2.37 (t, *J* = 8.2 Hz, 1H), 2.84 (s, 1H), 2.94–3.05 (m, 3H), 3.16 (dd, *J* = 6.0 Hz, *J* = 13.9 Hz, 2H), 3.27–3.34 (m, 3H), 3.35–3.44 (m, 3H), 3.87 (s, 3H), 4.66 (dd, *J* = 7.9 Hz, *J* = 14.1 Hz, 1H), 6.60 (d, *J* = 7.8 Hz, 1H), 6.86–6.97 (m, 5H), 7.00 (d, *J* = 7.7 Hz, 1H), 7.05–7.11 (m, 1H), 7.21–7.25 (m, 1H), 7.60–7.70 (m, 1H). ^13^C-NMR (CDCl_3_): δ 17.7, 23.7, 25.7, 29.1, 29.7, 30.7, 37.7, 44.9, 47.7, 49.5, 52.5, 54.4, 55.5, 111.4, 113.7, 116.3, 118.9, 121.2, 124.5, 125.1, 130.0, 138.9, 139.5, 152.1, 161.8, 163.8, 172.3, 176.8. HRMS calcd. for (M + H)^+^: C_30_H_42_FN_4_O_3_ 525.3241, found 525.3222.

*2-[(Cyclohexanecarbonyl)amino]-N-{[4-(2-methoxyphenyl)-piperazin-1-yl]propyl}-3-(4-fluoro-phenyl)propanamide* (**3h**): Product **3h** was obtained from resin **23**, 4-fluorobenzyl bromide, cyclo-hexanecarboxylic acid, and 2-methoxyphenylpiperazine as an amorphous light yellow solid (16.3 mg, 62% isolated yield) following chromatographic purification over silica gel with CH_2_Cl_2_–MeOH (93:7). Initial LC/MS purity 65%, *t*_R_ = 9.0 min; ^1^H-NMR (CDCl_3_): δ 1.13-1.27 (m, 3H), 1.28-1.41 (m, 2H), 1.59-1.66 (m, 1H), 1.67–1.82 (m, 6H), 1.98–2.05 (m, 0.4H), 2.05–2.14 (m, 1H), 2.37 (t, *J* = 8.2 Hz, 0.4H), 2.54–2.66 (m, 2H), 2.75–2.86 (m, 3H), 2.93–3.00 (m, 1H), 3.02–3.08 (m, 1H), 3.09–3.20 (m, 3H), 3.22–3.41 (m, 3H), 3.86 (s, 3H), 4.58 (dd, *J* = 7.3 Hz, *J* = 14.8 Hz, 1H), 6.48 (brs, 1H), 6.87 (d, *J* = 8.1 Hz, 1H), 6.91–6.99 (m, 4H), 7.00–7.07 (m, 1H), 7.12–7.20 (m, 2H), 7.49 (brs, 1H). ^13^C-NMR (CDCl_3_): δ 17.6, 24.4, 25.7, 29.2, 29.7, 30.7, 37.9, 45.1, 49.5, 53.1, 54.5, 55.4, 56.2, 111.3, 115.4, 118.5, 121.1, 123.6, 130.9, 132.5, 140.3, 152.2, 160.9, 162.8, 171.3, 176.3. HRMS calcd. for (M + H)^+^: C_30_H_42_FN_4_O_3_ 525.3241, found 525.3244.

*2-[(Benzoyl)amino]-N-{[4-(2-methoxyphenyl)-piperazin-1-yl]propyl}octanamide* (**3i**): Product **3i** was obtained from resin **23**, 1-iodohexane, benzoic acid, and 2-methoxyphenylpiperazine as light yellow oil (17.4 mg, 70% isolated yield) following chromatographic purification over silica gel with CH_2_Cl_2_–MeOH (93:7). Initial LC/MS purity 83%, *t*_R_ = 10.1 min; ^1^H-NMR (CDCl_3_): δ 0.81–0.89 (m, 3H), 1.21–1.41 (m, 8H), 1.71–1.83 (m, 1H), 1.84–1.98 (m, 3H), 1.99–2.06 (m, 0.4H), 2.35 (t, *J* = 8.2 Hz, 0.5H), 2.83 (s, 1H), 2.90–2.99 (m, 2H), 3.02–3.13 (m, 2H), 3.19–3.29 (m, 3H), 3.31–3.49 (m, 3H), 3.85 (s, 3H), 4.57 (dd, *J* = 7.7 Hz, *J* = 13.7 Hz, 1H), 6.82–6.97 (m, 3H), 7.01–7.10 (m, 1H), 7.29–7.36 (m, 1H), 7.40 (t, *J* = 7.5 Hz, 2H), 7.48 (t, *J* = 7.4 Hz, 1H), 7.86 (d, *J* = 7.3 Hz, 2H), 7.94 (brs, 1H). ^13^C-NMR (CDCl_3_): δ 14.0, 17.7, 22.5, 23.9, 25.8, 29.0, 30.7, 31.6, 32.6, 37.0, 48.4, 49.5, 52.8, 54.5, 55.4, 111.3, 118.7, 121.2, 124.1, 127.3, 128.6, 131.8, 133.6, 139.5, 152.2, 167.6, 173.1. HRMS calcd. for (M + H)^+^: C_29_H_43_N_4_O_3_ 495.3335, found 495.3337.

*2-[(Benzoyl)amino]-N-{[4-(2-methoxyphenyl)-piperazin-1-yl]propyl}-3(4-methoxyphenyl)-propan-amide* (**3j**): Product **3j** was obtained from resin **23**, 4-methoxybenzyl chloride, benzoic acid, and 2-methoxyphenylpiperazine as an amorphous light yellow solid (10.3 mg, 39% isolated yield) following chromatographic purification over silica gel with CH_2_Cl_2_–MeOH (93:7). Initial LC/MS purity 71%, *t*_R_ = 7.9 min; ^1^H-NMR (CDCl_3_): δ 1.72-1.87 (m, 2H), 1.96–2.09 (m, 1H), 2.37 (t, *J* = 8.2 Hz, 1H), 2.63–2.73 (m, 2H), 2.79–2.91 (m, 3H), 3.10–3.20 (m, 5H), 3.31–3.39 (m, 2H), 3.76 (s, 3H), 3.86 (s, 3H), 4.74 (dd, *J* = 7.2 Hz, *J* = 14.4 Hz, 1H), 6.83 (d, *J* = 8.6 Hz, 2H), 6.86–6.89 (m, 1H), 6.89–6.97 (m, 2H), 7.02–7.09 (m, 2H), 7.18 (d, *J* = 8.6 Hz, 2H), 7.37–7.45 (m, 3H), 7.46–7.51 (m, 1H), 7.74–7.79 (m, 2H). ^13^C-NMR (CDCl_3_): δ 17.7, 24.2, 29.6, 30.7, 37.9, 49.2, 49.4, 52.9, 55.2, 55.4, 55.6, 111.3, 114.1, 118.6, 121.2, 123.7, 127.1, 128.6, 128.7, 130.4, 131.7, 133.8, 152.2, 158.6, 167.1, 171.4. HRMS calcd. for (M + H)^+^: C_31_H_29_N_4_O_4_ 531.2971, found 531.2951.

*2-[(Benzoyl)amino]-N-{[4-(2-methoxyphenyl)-piperazin-1-yl]propyl}-3-(3-fluorophenyl)-propanamide* (**3k**): Product **3k** was obtained from resin **23**, 3-fluorobenzyl chloride, benzoic acid, and 2-methoxyphenylpiperazine as yellow oil (12.6 mg, 49% isolated yield) following chromatographic purification over silica gel with CH_2_Cl_2_–MeOH (93:7). Initial LC/MS purity 75%, *t*_R_ = 8.4 min; ^1^H- NMR (CDCl_3_): δ 1.76–1.93 (m, 2H), 1.96–2.08 (m, 1H), 2.37 (t, *J* = 8.2 Hz, 1H), 2.71–2.82 (m, 2H), 2.83–2.86 (m, 1H), 2.87–2.97 (m, 2H), 3.14–3.25 (m, 5H), 3.28–3.34 (m, 1H), 3.36–3.40 (m, 1H), 3.86 (s, 3H), 4.81 (dd, *J* = 7.2 Hz, *J* = 14.6 Hz, 1H), 6.86–6.89 (m, 1H), 6.89–6.96 (m, 3H), 6.97–7.01 (m, 1H), 7.02–7.08 (m, 2H), 7.16–7.22 (m, 1H), 7.23–7.26 (m, 1H), 7.37–7.43 (m, 2H), 7.45–7.51 (m, 1H), 7.59–7.70 (m, 1H), 7.73–7.80 (m, 2H). ^13^C-NMR (CDCl_3_): δ 17.7, 24.0, 29.6, 30.7, 38.3, 48.9, 49.4, 52.9, 55.3, 55.4, 111.3, 113.8, 113.9, 118.7, 121.2, 123.9, 125.1, 127.2, 128.6, 130.1, 131.8, 133.6, 139.4, 139.5, 152.2, 161.9, 163.8, 167.2, 171.3. HRMS calcd. for (M + H)^+^: C_30_H_36_FN_4_O_3_ 519.2771, found 519.2782.

*2-[(Benzoyl)amino]-N-{[4-(2-methoxyphenyl)-piperazin-1-yl]propyl}-3-(4-fluorophenyl)-propanamide* (**3l**): Product **3l** was obtained from resin **23**, 4-fluorobenzyl bromide, benzoic acid, and 2-methoxy-phenylpiperazine as brownish oil (19.7 mg, 76% isolated yield) following chromatographic purification over silica gel with CH_2_Cl_2_–MeOH (94:6). Initial LC/MS purity 86%, *t*_R_ = 8.3 min; ^1^H-NMR (CDCl_3_): δ 1.78–1.93 (m, 2H), 1.95–2.06 (m, 0.5H), 2.36 (t, *J* = 8.2 Hz, 0.5H), 2.75–2.88 (m, 3H), 2.93–3.04 (m, 2H), 3.07–3.16 (m, 2H), 3.17–3.32 (m, 5H), 3.34–3.41 (m, 1H), 3.85 (s, 3H), 4.71–4.92 (m, 1H), 6.85–6.91 (m, 2H), 7.01–7.09 (m, 1H), 7.19–7.25 (m, 2H), 7.37 (t, *J* = 7.6 Hz, 2H), 7.46 (t, *J* = 7.4 Hz, 1H), 7.77 (d, *J* = 7.4 Hz, 2H), 7.85 (brs, 1H). ^13^C-NMR (CDCl_3_): δ 17.6, 23.8, 29.6, 30.7, 37.5, 48.5, 49.5, 52.7, 55.4, 55.5, 111.3, 115.5, 118.7, 121.2, 124.1, 127.2, 128.5, 130.9, 131.9, 132.5, 133.4, 139.5, 152.2, 160.9, 162.8, 167.5, 171.8. HRMS calcd. for (M + H)^+^: C_30_H_36_FN_4_O_3_ 519.2771, found 519.2745.

*2-[(Quinolin-2-oyl)amino]-N-{[4-(2-methoxyphenyl)-piperazin-1-yl]propyl}octanamide* (**3m**): Product **3m** was obtained from resin **23**, 1-iodohexane, quinaldic acid, and 2-methoxyphenylpiperazine as brownish oil (18.4 mg, 67% isolated yield) following chromatographic purification over silica gel with CH_2_Cl_2_–MeOH (95:5). Initial LC/MS purity 80%, *t*_R_ = 12.1 min; ^1^H-NMR (CDCl_3_): δ 0.85 (t, *J* = 6.9 Hz, 3H), 1.24–1.30 (m, 4H), 1.32–1.49 (m, 4H), 1.78–1.92 (m, 1H), 1.94–2.10 (m, 3.5H), 2.37 (t, *J* = 8.1 Hz, 0.5H), 2.83 (s, 1H), 2.96–3.10 (m, 3H), 3.19–3.32 (m, 4H), 3.34–3.42 (m, 2H), 3.43–3.53 (m, 1H), 3.84 (s, 3H), 4.59 (dd, *J* = 7.3 Hz, *J* = 13.7 Hz, 1H), 6.82–6.96 (m, 3H), 6.99–7.09 (m, 1H), 7.57–7.71 (m, 2H), 7.76 (t, *J* = 8.2 Hz, 1H), 7.84 (d, *J* = 8.2 Hz, 1H), 8.14 (d, *J* = 8.5 Hz, 1H), 8.21–8.34 (m, 2H), 8.61–8.82 (m, 1H). ^13^C-NMR (CDCl_3_): δ 14.0, 17.6, 22.5, 25.8, 28.9, 30.7, 31.6, 32.3, 48.2, 49.5, 52.7, 54.2, 55.4, 111.3, 118.8, 121.2, 124.1, 127.7, 128.1, 129.4, 129.9, 130.2, 137.6, 139.4, 146.5, 148.9, 152.1, 164.9, 172.4. HRMS calcd. for (M + H)^+^: C_32_H_44_N_5_O_3_ 546.3444, found 546.3419.

*2-[(Quinolin-2-oyl)amino]-N-{[4-(2-methoxyphenyl)-piperazin-1-yl]propyl}-3-(4-methoxyphenyl)-propanamide* (**3n**): Product **3n** was obtained from resin **23**, 4-methoxybenzyl chloride, quinaldic acid, and 2-methoxyphenylpiperazine as brownish oil (10.5 mg, 36% isolated yield) following chromatographic purification over silica gel with CH_2_Cl_2_–MeOH (94:6). Initial LC/MS purity 66%, *t*_R_ = 9.9 min; ^1^H-NMR (CDCl_3_): δ 1.61–1.87 (m, 2H), 1.95–2.08 (m, 1H), 2.37 (t, *J* = 8.1 Hz, 1H), 2.56–2.67 (m, 2H), 2.77–2.88 (m, 3H), 3.08–3.15 (m, 2H), 3.16–3.28 (m, 3H), 3.32–3.35 (m, 1H), 3.36–3.39 (m, 1H), 3.77 (s, 3H), 3.85 (s, 3H), 4.76 (dd, *J* = 7.7 Hz, *J* = 14.4 Hz, 1H), 6.81–6.88 (m, 4H), 6.89–6.93 (m, 1H), 6.98–7.05 (m, 1H), 7.15–7.22 (m, 1H), 7.23–7.26 (m, 2H), 7.57–7.66 (m, 1H), 7.71–7.80 (m, 1H), 7.86 (d, *J* = 8.1 Hz, 1H), 8.10 (d, *J* = 8.4 Hz, 1H), 8.21 (d, *J* = 8.5 Hz, 1H), 8.27 (d, *J* = 8.5 Hz, 1H), 8.78 (d, *J* = 8.1 Hz, 1H). ^13^C-NMR (CDCl_3_): δ 17.7, 24.4, 29.6, 30.7, 37.7, 49.4, 52.9, 55.2, 55.4, 55.5, 56.1, 111.2, 114.0, 118.7, 121.1, 123.5, 127.7, 128.1, 128.8, 129.4, 129.9, 130.2, 130.5, 137.5, 146.5, 149.1, 152.2, 158.6, 164.5, 170.9. HRMS calcd. for (M + H)^+^: C_34_H_40_N_5_O_4_ 582.3080, found 582.3058.

*2-[(Quinolin-2-oyl)amino]-N-{[4-(2-methoxyphenyl)-piperazin-1-yl]propyl}-3-(3-fluorophenyl)-propanamide* (**3o**): Product **3o** was obtained from resin **23**, 3-fluorobenzyl chloride, quinaldic acid, and 2-methoxyphenylpiperazine as brownish oil (15.3 mg, 54% isolated yield) following chromatographic purification over silica gel with CH_2_Cl_2_–MeOH (93:7). Initial LC/MS purity 77%, *t*_R_ = 10.3 min; ^1^H- NMR (CDCl_3_): δ 1.88–2.12 (m, 3H), 2.37 (t, *J* = 8.1 Hz, 1H), 2.84 (s, 1H), 2.93–3.04 (m, 2H), 3.19–3.44 (m, 9H), 3.86 (s, 3H), 4.84 (dd, *J* = 7.0 Hz, *J* = 14.5 Hz, 1H), 6.85–6.98 (m, 4H), 7.02–7.10 (m, 2H), 7.13 (d, *J* = 7.7 Hz, 1H), 7.27-7.33 (m, 1H), 7.40–7.49 (m, 1H), 7.62 (t, *J* = 7.1 Hz, 1H), 7.73–7.81 (m, 1H), 7.85 (d, *J* = 8.1 Hz, 1H), 8.10 (d, *J* = 8.4 Hz, 1H), 8.17 (d, *J* = 8.5 Hz, 1H), 8.27 (d, *J* = 8.4 Hz, 1H), 8.76 (d, *J* = 7.8 Hz, 1H). ^13^C-NMR (CDCl_3_): δ 17.7, 23.7, 29.6, 30.7, 37.9, 47.8, 49.4, 52.5, 54.9, 55.4, 111.3, 114.1, 116.3, 118.5, 118.8, 121.2, 124.3, 125.2, 127.7, 128.1, 129.4, 129.9, 130.2, 137.5, 139.3, 146.5, 148.8, 152.1, 161.9, 162.7, 163.8, 164.8, 171.3. HRMS calcd. for (M + H)^+^: C_33_H_37_FN_5_O_3_ 570.2880, found 570.2853.

*2-[(Quinolin-2-oyl)amino]-N-{[4-(2-methoxyphenyl)-piperazin-1-yl]propyl}-3-(4-fluorophenyl)-propanamide* (**3p**): Product **3p** was obtained from resin **23**, 4-fluorobenzyl bromide, quinaldic acid, and 2-methoxyphenylpiperazine as brownish oil (22.1 mg, 78% isolated yield) following chromatographic purification over silica gel with CH_2_Cl_2_–MeOH (93:7). Initial LC/MS purity 85%, *t*_R_ = 10.2 min; ^1^H-NMR (CDCl_3_): δ 1.92–2.08 (m, 2.5H), 2.32–2.42 (m, 0.5H), 2.83 (s, 1H), 2.93–3.04 (m, 2H), 3.18–3.44 (m, 10H), 3.85 (s, 3H), 4.83 (dd, *J* = 7.3 Hz, *J* = 14.7 Hz, 1H), 6.84–6.95 (m, 3H), 6.96–7.02 (m, 2H), 7.03–7.09 (m, 1H), 7.28–7.36 (m, 2H), 7.47–7.56 (m, 1H), 7.61 (t, *J* = 7.4 Hz, 1H), 7.76 (t, *J* = 7.1 Hz, 1H), 7.85 (d, *J* = 8.2 Hz, 1H), 8.09 (d, *J* = 8.5 Hz, 1H), 8.17 (d, *J* = 8.5 Hz, 1H), 8.26 (d, *J* = 8.5 Hz, 1H), 8.74 (d, *J* = 7.8 Hz, 1H). ^13^C-NMR (CDCl_3_): δ 17.7, 23.6, 29.6, 30.7, 36.5, 37.5, 47.7, 49.4, 52.5, 54.6, 55.2, 55.4, 111.3, 115.4, 118.6, 118.9, 121.2, 124.3, 127.7, 128.1, 129.4, 129.9, 130.2, 131.0, 132.5, 139.1, 146.5, 148.9, 152.1, 160.9, 162.4, 162.9, 164.7, 171.5. HRMS calcd. for (M + H)^+^: C_33_H_37_FN_5_O_3_ 570.2880, found 570.2870.

*2-[(Cyclopropanecarbonyl)amino]-N-{[4-(3-chlorophenyl)-piperazin-1-yl]propyl}octanamide* (**3q**): Product **3q** was obtained from resin **23**, 1-iodohexane, cyclopropanecarboxylic acid, and 3-chloro-phenylpiperazine as brownish oil (11.2 mg, 48% isolated yield) following chromatographic purification over silica gel with CH_2_Cl_2_–MeOH (93:7). Initial LC/MS purity 68%, *t*_R_ = 9.7 min; ^1^H- NMR (CDCl_3_): δ 0.66–0.79 (m, 2H), 0.81–0.97 (m, 5H), 1.18–1.37 (m, 8.6H), 1.38–1.52 (m, 1H), 1.56–1.70 (m, 1H), 1.76–1.84 (m, 1H), 1.84–1.93 (m, 1.6H), 1.96–2.08 (m, 0.4H), 2.37 (t, *J* = 8.1 Hz, 0.4H), 2.72–2.83 (m, 2H), 2.84 (s, 1H), 2.87–3.01 (m, 3H), 3.27–3.44 (m, 6H), 4.34 (dd, *J* = 7.5 Hz, *J* = 14.1 Hz, 1H), 6.62 (brs, 1H), 6.75–6.81 (m, 1H), 6.83–6.90 (m, 2H), 7.15–7.22 (m, 1H), 7.39–7.57 (m, 1H). ^13^C-NMR (CDCl_3_): δ 7.4, 7.5, 14.0, 14.6, 17.7, 22.5, 24.5, 25.6, 29.0, 31.6, 32.6, 47.7, 49.5, 52.4, 53.9, 55.7, 114.4, 116.5, 120.5, 130.3, 135.1, 151.4, 172.7, 174.1. HRMS calcd. for (M + H)^+^: C_25_H_40_ClN_4_O_2_ 463.2840, found 463.2841.

*2-[(Cyclopropanecarbonyl)amino]-N-{[4-(3-chlorophenyl)-piperazin-1-yl]propyl}-3(4-methoxy-phenyl)propanamide* (**3r**): Product **3r** was obtained from resin **23**, 4-methoxybenzyl chloride, cyclopropanecarboxylic acid, and 3-chlorophenyl piperazine as yellow oil (10.2 mg, 41% isolated yield) following chromatographic purification over silica gel with CH_2_Cl_2_–MeOH (92:8). Initial LC/MS purity 60%, *t*_R_ = 7.7 min; ^1^H-NMR (CDCl_3_): δ 0.67–0.79 (m, 2H), 0.85–0.94 (m, 2H), 1.39–1.44 (m, 1H), 1.71–1.83 (m, 2H), 1.97–2.08 (m, 1H), 2.37 (t, *J* = 8.2 Hz, 1H), 2.57–2.64 (m, 2H), 2.75–2.82 (m, 2H), 2.83–2.85 (m, 1H), 2.97–3.01 (m, 1H), 3.24–3.30 (m, 5H), 3.36–3.41 (m, 1H), 3.77 (s, 3H), 4.45–4.68 (m, 1H), 6.64 (brs, 1H), 6.77 (d, *J* = 7.4 Hz, 1H), 6.82 (d, *J* = 8.4 Hz, 2H), 6.87 (d, *J* = 7.0 Hz, 2H), 7.13 (d, *J* = 8.4 Hz, 2H), 7.18 (t, *J* = 8.3 Hz, 1H). ^13^C-NMR (CDCl_3_): δ 7.4, 7.5, 13.5, 14.6, 17.7, 29.6, 30.7, 37.7, 47.7, 49.5, 52.3, 55.3, 113.9, 114.4, 116.4, 120.4, 128.8, 130.3, 130.4, 135.1, 151.4, 158.5, 171.6, 173.8. HRMS calcd. for (M + H)^+^: C_27_H_36_ClN_4_O_3_ 499.2476, found 499.2467.

*2-[(Cyclopropanecarbonyl)amino]-N-{[4-(3-chlorophenyl)-piperazin-1-yl]propyl}-3-(3-fluorophenyl)-propanamide* (**3s**): Product **3s** was obtained from resin **23**, 3-fluorobenzyl chloride, cyclo-propanecarboxylic acid, and 3-chlorophenylpiperazine as light yellow oil (8.0 mg, 33% isolated yield) following chromatographic purification over silica gel with CH_2_Cl_2_–MeOH (93:7). Initial LC/MS purity 57%, *t*_R_ = 8.2 min; ^1^H-NMR (CDCl_3_): δ 0.67–0.81 (m, 2H), 0.86–0.96 (m, 2H), 1.37–1.47 (m, 1H), 1.77 (brs, 2H), 2.57–2.89 (m, 5H), 3.04–3.12 (m, 2H), 3.22–3.41 (m, 7H), 4.47–4.72 (m, 1H), 6.55 (brs, 1H), 6.74–6.81 (m, 1H), 6.85–6.89 (m, 2H), 6.91–6.96 (m, 2H), 7.00 (d, *J* = 7.7 Hz, 1H), 7.19 (t, *J* = 8.4 Hz, 2H), 7.23–7.26 (m, 1H). ^13^C-NMR (CDCl_3_): δ 7.5, 7.6, 14.6, 24.4, 38.3, 47.9, 52.5, 54.9, 56.0, 113.7, 114.3, 116.2, 116.4, 120.3, 125.2, 130.0, 130.2, 135.1, 139.5, 151.5, 161.8, 163.8, 171.4, 173.8. HRMS calcd. for (M + H)^+^: C_26_H_33_ClFN_4_O_2_ 487.2276, found 487.2299.

*2-[(Cyclopropanecarbonyl)amino]-N-{[4-(3-chlorophenyl)-piperazin-1-yl]propyl}-3-(4-fluorophenyl)-propanamide* (**3t**): Product **3t** was obtained from resin **23**, 4-fluorobenzyl bromide, cyclo-propanecarboxylic acid, and 3-chlorophenylpiperazine as brown oil (9.9 mg, 41% isolated yield) following chromatographic purification over silica gel with CH_2_Cl_2_–MeOH (93:7). Initial LC/MS purity 73%, *t*_R_ = 8.1 min; ^1^H-NMR (CDCl_3_): δ 0.67–0.78 (m, 2H), 0.83–0.94 (m, 2H), 1.37–1.47 (m, 1H), 1.69–1.85 (m, 2H), 2.58–2.73 (m, 2H), 2.75–2.94 (m, 4H), 2.98–3.07 (m, 2H), 3.21–3.39 (m, 6H), 4.58 (dd, *J* = 7.3 Hz, *J* = 14.7 Hz, 1H), 6.63 (brs, 1H), 6.74–6.79 (m, 1H), 6.84–6.91 (m, 2H), 6.94–7.01 (m, 2H), 7.10–7.26 (m, 4H). ^13^C-NMR (CDCl_3_): δ 7.4, 7.6, 14.6, 24.3, 37.7, 47.6, 52.3, 55.1, 55.6, 114.5, 115.4, 116.5, 120.6, 130.3, 130.9, 132.6, 135.1, 151.3, 160.9, 162.8, 171.4, 173.9. HRMS calcd. for (M + H)^+^: C_26_H_33_ClFN_4_O_2_ 487.2276, found 487.2292.

*2-[(Cyclohexanecarbonyl)amino]-N-{[4-(3-chlorophenyl)-piperazin-1-yl]propyl}octanamide* (**3u**): Product **3u** was obtained from resin **23**, 1-iodohexane, cyclohexanecarboxylic acid, and 3-chlorophenyl piperazine as brown oil (10.9 mg, 43% isolated yield) following chromatographic purification over silica gel with CH_2_Cl_2_–MeOH (94:6). Initial LC/MS purity 66%, *t*_R_ = 12.2 min; ^1^H-NMR (CDCl_3_): δ 0.76–0.92 (m, 3H), 1.16–1.33 (m, 11H), 1.35–1.47 (m, 2H), 1.55–1.69 (m, 2H), 1.71-1.95 (m, 7H), 1.97–2.07 (m, 0.5H), 2.09–2.22 (m, 1H), 2.38 (t, *J* = 8.0 Hz, 0.5H), 2.78–2.86 (m, 2H), 2.89–3.04 (m, 3H), 3.25–3.45 (m, 6H), 4.15–4.44 (m, 1H), 6.38 (brs, 1H), 6.78 (d, *J* = 8.1 Hz, 1H), 6.82–6.93 (m, 2H), 7.18 (t, *J* = 8.3 Hz, 1H), 7.53 (brs, 1H). ^13^C-NMR (CDCl_3_): δ 14.0, 17.7, 22.5, 25.5, 25.7, 28.9, 29.3, 29.8, 31.6, 32.4, 37.3, 45.2, 47.6, 49.5, 52.4, 53.5, 55.5, 114.5, 116.6, 120.6, 130.3, 135.1, 151.3, 172.8, 176.7. HRMS calcd. for (M + H)^+^: C_28_H_46_ClN_4_O_2_ 505.3309, found 505.3318.

*2-[(Cyclohexanecarbonyl)amino]-N-{[4-(3-chlorophenyl)-piperazin-1-yl]propyl}-3(4-methoxyphenyl)-propanamide* (**3v**): Product **3v** was obtained from resin **23**, 4-methoxybenzyl chloride, cyclo-hexanecarboxylic acid, and 3-chlorophenylpiperazine as an amorphous off-white solid (6.9 mg, 26% isolated yield) following chromatographic purification over silica gel with CH_2_Cl_2_–MeOH (93:7). Initial LC/MS purity 55%, *t*_R_ = 10.1 min; ^1^H-NMR (CDCl_3_): δ 1.17–1.26 (m, 3H), 1.31–1.41 (m, 2H), 1.61–1.67 (m, 1H), 1.71–1.84 (m, 7H), 1.98–2.05 (m, 0.6H), 2.07–2.15 (m, 1H), 2.37 (t, *J* = 8.2 Hz, 0.6H), 2.59–2.71 (m, 3H), 2.84 (s, 1H), 2.98 (d, *J* = 6.7 Hz, 2H), 3.24–3.35 (m, 6H), 3.78 (s, 3H), 4.51 (dd, *J* = 7.1 Hz, *J* = 14.3 Hz, 1H), 6.22 (brs, 1H), 6.75–6.79 (m, 1H), 6.82 (d, *J* = 8.5 Hz, 2H), 6.86–6.89 (m, 2H), 6.96–7.07 (m, 1H), 7.12 (d, *J* = 8.6 Hz, 2H), 7.16–7.22 (m, 1H). ^13^C-NMR (CDCl_3_): δ 17.7, 24.4, 25.5, 25.7, 29.3, 29.7, 30.7, 37.7, 45.1, 49.4, 52.4, 54.8, 55.3, 113.9, 114.4, 116.4, 120.4, 128.8, 130.2, 130.4, 135.1, 151.4, 158.5, 171.5, 176.2. HRMS calcd. for (M + H)^+^: C_30_H_42_ClN_4_O_3_ 541.2945, found 541.2943.

*2-[(Cyclohexanecarbonyl)amino]-N-{[4-(3-chloroyphenyl)-piperazin-1-yl]propyl}-3-(3-fluorophenyl)-propanamide* (**3w**): Product **3w** was obtained from resin **23**, 3-fluorobenzyl chloride, cyclo-hexanecarboxylic acid, and 3-chlorophenylpiperazine as an amorphous light yellow solid (8.5 mg, 32% isolated yield) following chromatographic purification over silica gel with CH_2_Cl_2_–MeOH (94:6). Initial LC/MS purity 54%, *t*_R_ = 10.6 min; ^1^H-NMR (CDCl_3_): δ 1.17–1.28 (m, 3H), 1.29–1.41 (m, 2H), 1.62–1.81 (m, 7H), 1.99–2.14 (m, 2H), 2.37 (t, *J* = 8.0 Hz, 1H), 2.49–2.59 (m, 2H), 2.62–2.73 (m, 3H), 2.84 (s, 1H), 2.96–3.09 (m, 2H), 3.18–3.25 (m, 3H), 3.27–3.31 (m, 1H), 3.38 (t, *J* = 7.0 Hz, 1H), 4.48–4.64 (m, 1H), 6.32 (brs, 1H), 6.77 (d, *J* = 8.2 Hz, 1H), 6.83–6.89 (m, 2H), 6.90–6.96 (m, 2H), 6.98 (d, *J* = 7.6 Hz, 1H), 7.18 (t, *J* = 8.0 Hz, 1H), 7.21–7.26 (m, 1H). ^13^C-NMR (CDCl_3_): δ 17.7, 24.7, 25.5, 25.6, 29.3, 29.7, 30.7, 38.3, 45.1, 48.1, 49.5, 52.6, 54.4, 56.2, 113.8, 113.9, 114.2, 116.2, 116.3, 120.0, 125.1, 130.2, 139.3, 151.7, 161.8, 163.8, 170.9, 176.2. HRMS calcd. for (M + H)^+^: C_29_H_39_ClFN_4_O_2_ 529.2746, found 529.2736.

*2-[(Cyclohexanecarbonyl)amino]-N-{[4-(3-chloroyphenyl)-piperazin-1-yl]propyl}-3-(4-fluorophenyl)-propanamide (**3x**): * Product **3x** was obtained from resin **23**, 4-fluorobenzyl bromide, cyclo-hexanecarboxylic acid, and 3-chlorophenylpiperazine as an amorphous yellow solid (11.7 mg, 44% isolated yield) following chromatographic purification over silica gel with CH_2_Cl_2_–MeOH (93:7). Initial LC/MS purity 72%, *t*_R_ = 10.5 min; ^1^H-NMR (CDCl_3_): δ 1.11–1.28 (m, 3H), 1.29–1.41 (m, 2H), 1.60–1.67 (m, 1H), 1.69–1.82 (m, 5H), 1.97–2.04 (m, 0.5H), 2.05–2.14 (m, 1H), 2.37 (t, *J* = 8.2 Hz, 0.5H), 2.59–2.73 (m, 2H), 2.75–2.92 (m, 4H), 2.97 (dd, *J* = 7.1 Hz, *J* = 13.7 Hz, 1H), 3.06 (dd, *J* = 7.2 Hz, *J* = 13.6 Hz, 1H), 3.23–3.35 (m, 5H), 3.35–3.40 (m, 1H), 4.55 (dd, *J* = 7.1 Hz, *J* = 14.4 Hz, 1H), 6.36 (d, *J* = 6.0 Hz, 1H), 6.77 (d, *J* = 8.3 Hz, 1H), 6.83–6.90 (m, 2H), 6.96 (t, *J* = 8.5 Hz, 2H), 7.11–7.22 (m, 3H), 7.24–7.26 (m, 1H). ^13^C-NMR (CDCl_3_): δ 17.7, 24.4, 25.7, 29.2, 29.7, 30.7, 37.7, 45.1, 47.7, 49.5, 52.4, 54.6, 55.6, 114.4, 115.3, 116.5, 120.5, 130.3, 130.9, 132.6, 135.1, 151.3, 160.9, 162.8, 171.4, 176.4. HRMS calcd. for (M + H)^+^: C_29_H_39_ClFN_4_O_2_ 529.2746, found 529.2741.

*2-[(Benzoyl)amino]-N-{[4-(3-chlorophenyl)-piperazin-1-yl]propyl}octanamide* (**3y**): Product **3y** was obtained from resin **23**, 1-iodohexane, benzoic acid, and 3-chlorophenylpiperazine as brown oil (10.3 mg, 41% isolated yield) following chromatographic purification over silica gel with CH_2_Cl_2_–MeOH (94:6). Initial LC/MS purity 68%, *t*_R_ = 11.5 min; ^1^H-NMR (CDCl_3_): δ 0.78–0.89 (m, 3H), 1.10–1.41 (m, 9H), 1.68–1.81 (m, 1H), 1.84–1.94 (m, 2H), 1.97–2.08 (m, 0.4H), 2.37 (t, *J* = 8.1 Hz, 0.4H), 2.70–2.80 (m, 2H), 2.81–2.93 (m, 3H), 3.23–3.46 (m, 6H), 4.38–4.69 (m, 1H), 6.76 (d, *J* = 8.6 Hz, 1H), 6.86 (d, *J* = 8.4 Hz, 2H), 7.05 (brs, 1H), 7.17 (t, *J* = 8.0 Hz, 1H), 7.42 (t, *J* = 7.5 Hz, 2H), 7.49 (t, *J* = 7.3 Hz, 1H), 7.59 (brs, 1H), 7.83 (d, *J* = 7.4 Hz, 2H). ^13^C-NMR (CDCl_3_): δ 14.0, 17.7, 22.5,, 29.0, 31.6, 32.7, 37.8, 47.7, 52.5, 54.2, 55.9, 114.4, 116.4, 120.4, 127.2, 128.6, 130.2, 131.8, 133.7, 135.1, 151.4, 167.4, 172.4. HRMS calcd. for (M + H)^+^: C_28_H_40_ClN_4_O_2_ 499.2840, found 499.2840.

*2-[(Benzoyl)amino]-N-{[4-(3-chlorophenyl)-piperazin-1-yl]propyl}-3(4-methoxyphenyl)-propanamide* (**3z**): Product **3z** was obtained from resin **23**, 4-methoxybenzyl chloride, benzoic acid, and 3-chlorophenyl piperazine as an amorphous white solid (5.2 mg, 19% isolated yield) following chromatographic purification over silica gel with CH_2_Cl_2_–MeOH (94:6). Initial LC/MS purity 59%, *t*_R_ = 9.4 min; ^1^H -MR (CDCl_3_): δ 1.64–1.75 (m, 2H), 2.43–2.73 (m, 6H), 3.05–3.21 (m, 6H), 3.26–3.39 (m, 2H), 3.78 (s, 3H), 4.69 (dd, *J* = 7.5 Hz, *J* = 13.7 Hz, 1H), 6.74 (d, *J* = 7.4 Hz, 1H), 6.79–6.87 (m, 4H), 6.92 (d, *J* = 6.0 Hz, 1H), 7.08 (brs, 1H), 7.14–7.21 (m, 3H), 7.39 (t, *J* = 7.6 Hz, 2H), 7.48 (t, *J* = 7.4 Hz, 1H), 7.74 (d, *J* = 7.3 Hz, 2H). ^13^C-NMR (CDCl_3_): δ 24.6, 37.9, 38.5, 48.2, 52.7, 55.3, 55.6, 56.6, 114.1, 116.1, 119.9, 127.1, 128.6, 128.7, 130.1, 130.4, 131.8, 133.7, 135.0, 151.7, 158.7, 167.0, 170.9. HRMS calcd. for (M + H)^+^: C_30_H_36_ClN_4_O_3_ 535.2476, found 535.2470.

*2-[(Benzoyl)amino]-N-{[4-(3-chlorophenyl)-piperazin-1-yl]propyl}-3-(3-fluorophenyl)-propanamide* (**3aa**): Product **3aa** was obtained from resin **23**, 3-fluorobenzyl chloride, benzoic acid, and 3-chlorophenylpiperazine as light yellow oil (9.2 mg, 35% isolated yield) following chromatographic purification over silica gel with CH_2_Cl_2_–MeOH (94:6). Initial LC/MS purity 61%, *t*_R_ = 9.9 min; ^1^H- NMR (CDCl_3_): δ 1.61–1.77 (m, 2H), 1.96–2.07 (m, 1H), 2.37 (t, *J* = 8.1 Hz, 1H), 2.49–2.54 (m, 1H), 2.55–2.63 (m, 2H), 2.64–2.69 (m, 1H), 2.84 (s, 1H), 3.14–3.19 (m, 4H), 3.25–3.40 (m, 3H), 4.64–4.85 (m, 1H), 6.75 (d, *J* = 7.6 Hz, 1H), 6.85 (d, *J* = 7.4 Hz, 2H), 6.91–7.01 (m, 2H), 7.02–7.09 (m, 2H)), 7.14–7.21 (m, 1H), 7.22–7.26 (m, 1H), 7.29–7.36 (m, 1H), 7.39 (t, *J* = 7.6 Hz, 2H), 7.46–7.52 (m, 1H), 7.75 (d, *J* = 7.8 Hz, 2H). ^13^C-NMR (CDCl_3_): δ 17.7, 14.5, 19.6, 30.7, 38.4, 48.1, 49.5, 52.6, 55.3, 56.4, 113.9, 114.0, 114.2, 116.2, 116.4, 119.9, 125.1, 127.1, 128.6, 130.1, 131.9, 133.5, 135.0, 139.4, 151.7, 161.9, 163.8, 167.2, 170.8. HRMS calcd. for (M + H)^+^: C_29_H_33_ClFN_4_O_2_ 523.2276, found 523.2283.

*2-[(Benzoyl)amino]-N-{[4-(3-chlorophenyl)-piperazin-1-yl]propyl}-3-(4-fluorophenyl)-propanamide* (**3ab**): Product **3ab** was obtained from resin **23**, 4-fluorobenzyl bromide, benzoic acid, and 3-chloro-phenylpiperazine as an amorphous light yellow solid (14.2 mg, 54% isolated yield) following chromatographic purification over silica gel with CH_2_Cl_2_–MeOH (94:6). Initial LC/MS purity 73%, *t*_R_ = 9.8 min; ^1^H-NMR (CD_3_OD/CDCl_3_): δ 1.74–1.85 (m, 2H), 1.97–2.07 (m, 1H), 2.37 (t, *J* = 8.1 Hz, 1H), 2.63–2.76 (m, 2H), 2.82–2.92 (m, 3H), 3.09 (dd, *J* = 7.2 Hz, *J* = 13.5 Hz, 1H), 3.16 (dd, *J* = 7.4 Hz, *J* = 13.7 Hz, 1H), 3.23–3.34 (m, 5H), 4.63–4.84 (m, 1H), 6.72–6.81 (m, 1H), 6.82–6.92 (m, 2H), 6.97 (t, *J* = 8.6 Hz, 2H), 7.09–7.26 (m, 3H), 7.41 (t, *J* = 7.6 Hz, 2H), 7.47–7.53 (m, 1H), 7.76 (d, *J* = 7.8 Hz, 2H). ^13^C-NMR (CD_3_OD/CDCl_3_): δ 17.6, 24.3, 29.6, 30.7, 37.5, 47.5, 52.3, 55.4, 114.5, 115.4, 115.5, 116.6, 120.6, 127.2, 128.6, 130.3, 130.8, 130.9, 132.0, 132.4, 133.3, 135.1, 151.2, 160.9, 162.9, 167.6, 168.8. HRMS calcd. for (M + H)^+^: C_29_H_33_ClFN_4_O_2_ 523.2276, found 523.2252.

*2-[(Quinolin-2-oyl)amino]-N-{[4-(3-chloroyphenyl)-piperazin-1-yl]propyl}octanamide* (**3ac**): Product **3ac** was obtained from resin **23**, 1-iodohexane, quinaldic acid, and 3-chlorophenylpiperazine as brown oil (12.4 mg, 45% isolated yield) following chromatographic purification over silica gel with CH_2_Cl_2_–MeOH (95:5). Initial LC/MS purity 72%, *t*_R_ = 13.3 min; ^1^H-NMR (CDCl_3_): δ 0.86 (t, *J* = 6.9 Hz, 3H), 1.24–1.32 (m, 5H), 1.34–1.51 (m, 4H), 1.79–1.93 (m, 1H), 1.96–2.09 (m, 3H), 2.84 (s, 1H), 2.92–3.19 (m, 4H), 3.33–3.48 (m, 6H), 4.53 (dd, *J* = 7.6 Hz, 13.7 Hz, 1H), 6.72 (dd, *J* = 2.1 Hz, *J* = 8.3 Hz, 1H), 6.78–6.84 (m, 1H), 6.89 (d, *J* = 7.9 Hz, 1H), 7.17 (t, *J* = 8.1 Hz, 1H), 7.39 (brs, 1H), 7.58–7.66 (m, 1H), 7.72–7.81 (m, 1H), 7.86 (d, *J* = 8.1 Hz, 1H), 8.14 (d, *J* = 8.4 Hz, 1H), 8.20 (d, *J* = 8.5 Hz, 1H), 8.28 (d, *J* = 8.5 Hz, 1H), 8.63 (d, *J* = 7.5 Hz, 1H). ^13^C-NMR (CDCl_3_): δ 14.0, 17.7, 22.5, 25.9, 28.9, 31.6, 32.1, 36.9, 46.9, 52.1, 54.3, 55.2, 114.6, 116.7, 118.6, 120.9, 127.7, 128.2, 129.4, 129.9, 130.3, 135.1, 137.6, 146.5, 148.9, 150.9, 164.9, 172.4. HRMS calcd. for (M + H)^+^: C_31_H_41_ClN_5_O_2_ 550.2949, found 550.2936.

*2-[(Quinolin-2-oyl)amino]-N-{[4-(3-chlorophenyl)-piperazin-1-yl]propyl}-3-(4-methoxyphenyl)-propanamide* (**3ad**): Product **3ad** was obtained from resin **23**, 4-methoxybenzyl chloride, quinaldic acid, and 3-chlorophenylpiperazine as brown oil (8.0 mg, 27% isolated yield) following chromatographic purification over silica gel with CH_2_Cl_2_–MeOH (94:6). Initial LC/MS purity 58%, *t*_R_ = 11.3 min; ^1^H-NMR (CDCl_3_): δ 1.62–1.73 (m, 2H), 1.97–2.06 (m, 1H), 2.37 (t, *J* = 8.2 Hz, 1H), 2.42–2.47 (m, 1H), 2.48–2.59 (m, 3H), 2.84 (s, 1H), 2.97–3.07 (m, 3H), 3.17 (dd, *J* = 7.6 Hz, *J* = 13.7 Hz, 1H), 3.26 (dd, *J* = 5.8 Hz, *J* = 13.8 Hz, 1H), 3.32–3.39 (m, 2H), 3.78 (s, 3H), 4.76 (dd, *J* = 7.5 Hz, *J* = 14.0 Hz, 1H), 6.62 (d, *J* = 8.3 Hz, 1H), 6.69 (brs, 1H), 6.79 (d, *J* = 7.4 Hz, 1H), 6.86 (d, *J* = 8.4 Hz, 2H), 7.04–7.14 (m, 2H), 7.22–7.26 (m, 2H), 7.61 (t, *J* = 7.5 Hz, 1H), 7.69–7.77 (m, 1H), 7.84 (d, *J* = 8.2 Hz, 1H), 8.05 (d, *J* = 8.5 Hz, 1H), 8.16–8.28 (m, 2H), 8.75 (d, *J* = 7.9 Hz, 1H). ^13^C-NMR (CDCl_3_): δ 17.7, 24.7, 29.6, 30.7, 37.5, 47.9, 49.5, 52.8, 55.4, 113.8, 114.1, 115.7, 118.7, 127.7, 128.2, 128.8, 129.4, 129.8, 130.0, 130.2, 130.6, 134.9, 137.6, 146.4, 148.9, 156.6, 158.7, 164.6, 170.7. HRMS calcd. for (M + H)^+^: C_33_H_37_ClN_5_O_3_ 586.2585, found 586.2600.

*2-[(Quinolin-2-oyl)amino]-N-{[4-(3-chlorophenyl)-piperazin-1-yl]propyl}-3-(3-fluorophenyl)-propanamide* (**3ae**): Product **3ae** was obtained from resin **23**, 3-fluorobenzyl chloride, quinaldic acid, and 3-chlorophenylpiperazine as brown oil (9.4 mg, 33% isolated yield) following chromatographic purification over silica gel with CH_2_Cl_2_–MeOH (95:5). Initial LC/MS purity 59%, t_R_ = 11.6 min; ^1^H- NMR (CDCl_3_): δ 1.58–1.70 (m, 2H), 1.95–2.08 (m, 1H), 2.35–2.42 (m, 2H), 2.43–2.50 (m, 3H), 2.84 (s, 1H), 2.94–3.03 (m, 3H), 3.21–3.27 (m, 1H), 3.29–3.40 (m, 3H), 4.81 (dd, *J* = 7.9 Hz, *J* = 14.0 Hz, 1H), 6.92–7.00 (m, 1H), 7.04–7.13 (m, 3H), 7.17–7.22 (m, 1H), 7.27–7.32 (m, 1H), 7.57–7.64 (m, 1H), 7.71–7.77 (m, 1H), 7.84 (d, *J* = 8.2 Hz, 1H), 8.06 (d, *J* = 8.5 Hz, 1H), 8.15–8.28 (m, 2H), 8.73 (d, *J* = 8.4 Hz, 1H). ^13^C-NMR (CDCl_3_): δ 17.7, 24.7, 29.6, 30.7, 38.1, 39.2, 48.1, 52.9, 54.9, 57.0, 113.6, 113.9, 114.0, 115.5, 116.4, 116.6, 118.6, 119.3, 125.4, 127.7, 128.2, 129.4, 129.8, 129.9, 130.3, 134.9, 137.6, 139.4, 146.4, 148.8, 151.7, 161.9, 163.9, 164.6, 170.2. HRMS calcd. for (M + H)^+^: C_32_H_34_ClFN_5_O_2_ 574.2385, found 574.2402.

*2-[(Quinolin-2-oyl)amino]-N-{[4-(3-chlorophenyl)-piperazin-1-yl]propyl}-3-(4-fluorophenyl)-propanamide (***3af**)*.* Product **3af** was obtained from resin **23**, 4-fluorobenzyl bromide, quinaldic acid, and 3-chlorophenylpiperazine as brown oil (15.5 mg, 54% isolated yield) following chromatographic purification over silica gel with CH_2_Cl_2_–MeOH (93:7). Initial LC/MS purity 72%, *t*_R_ = 11.5 min; ^1^H- NMR (CDCl_3_): δ 1.84–1.98 (m, 2H), 1.98–2.05 (m, 0.5H), 2.37 (t, *J* = 8.1 Hz, 0.5H), 2.79–3.17 (m, 5H), 3.20–3.44 (m, 8H), 4.79 (dd, *J* = 7.2 Hz, *J* = 14.7 Hz, 1H), 6.71 (dd, *J* = 2.1 Hz, *J* = 8.3 Hz, 1H), 6.78–6.84 (m, 1H), 6.89 (dd, *J* = 1.1 Hz, *J* = 7.9 Hz, 1H), 6.96–7.04 (m, 2H), 7.17 (t, *J* = 8.1 Hz, 1H), 7.27–7.40 (m, 3H), 7.57–7.66 (m, 1H), 7.72–7.81 (m, 1H), 7.85 (d, *J* = 8.1 Hz, 1H), 8.07 (d, *J* = 8.5 Hz, 1H), 8.16 (d, *J* = 8.5 Hz, 1H), 8.26 (d, *J* = 8.5 Hz, 1H), 8.72 (d, *J* = 7.8 Hz, 1H). ^13^C-NMR (CDCl_3_): δ 17.7, 23.9, 29.6, 30.7, 37.1, 37.4, 46.9, 49.4, 52.0, 55.2, 114.5, 115.4, 115.6, 116.7, 118.5, 120.9, 127.7, 128.2, 129.4, 129.8, 130.3, 131.0, 132.4, 135.1, 137.6, 146.5, 148.8, 150.8, 160.9, 162.9, 164.8, 171.2. HRMS calcd. for (M + H)^+^: C_32_H_34_ClFN_5_O_2_ 574.2385, found 574.2376.
